# Brain morphology predicts social intelligence in wild cleaner fish

**DOI:** 10.1038/s41467-020-20130-2

**Published:** 2020-12-21

**Authors:** Zegni Triki, Yasmin Emery, Magda C. Teles, Rui F. Oliveira, Redouan Bshary

**Affiliations:** 1 Institute of Biology, University of Neuchâtel, Emile-Argand 11, 2000 Neuchâtel, Switzerland; 2 Department of Zoology, Stockholm University, Svante Arrheniusväg 18 B, Stockholm, Sweden; 3 Instituto Gulbenkian de Ciência, Rua da Quinta Grande, 6, 2780-156 Oeiras, Portugal; 4 ISPA – Instituto Universitário, Rua Jardim do Tabaco 34, 1149-041 Lisboa, Portugal

**Keywords:** Behavioural ecology, Social evolution, Social behaviour

## Abstract

It is generally agreed that variation in social and/or environmental complexity yields variation in selective pressures on brain anatomy, where more complex brains should yield increased intelligence. While these insights are based on many evolutionary studies, it remains unclear how ecology impacts brain plasticity and subsequently cognitive performance within a species. Here, we show that in wild cleaner fish (*Labroides dimidiatus*), forebrain size of high-performing individuals tested in an ephemeral reward task covaried positively with cleaner density, while cerebellum size covaried negatively with cleaner density. This unexpected relationship may be explained if we consider that performance in this task reflects the decision rules that individuals use in nature rather than learning abilities: cleaners with relatively larger forebrains used decision-rules that appeared to be locally optimal. Thus, social competence seems to be a suitable proxy of intelligence to understand individual differences under natural conditions.

## Abstract (web summary)

The causes and consequences of social intelligence are challenging to establish. A study on wild cleaner fish reports that large forebrains enable individuals to score higher in a social competence test, suggesting forebrain size is important for complex social decision-making.

## Introduction

Vertebrate species show a large variation in brain size^1^, which can be the result of selective forces emerging from the ecological complexity (i.e. social or environmental) of a species^2,3^. Comparative studies suggest that larger brains confer better cognitive abilities^4–8^. Indeed, the artificial selection on brain size can result in large-brained populations with higher cognitive abilities, such as enhanced learning in up-selected mice^9^, as well as in up-selected guppies^10,11^. However, natural selection acting on existing genetic variation does not represent the only way for individuals of a species to become well adapted to their environment. Phenotypic plasticity, i.e. the interactions between an individual’s genome and its environment, provides an alternative mechanism by which to achieve more fine-tuned adaptations to local conditions^12,13^. Thus, exploring the extent to which small-scale variation in ecological complexity may affect brain plasticity and thereby cognitive performance within a population, can complement the interspecific comparative approach^14–17^. Yet, assessing simultaneously the ecological factors, brain morphology, and cognitive performance at the intraspecific level has been rarely done.

Fishes are a suitable clade to study the links between ecology, brain plasticity and cognitive performance^18^ since there is solid evidence of brain plasticity in fish as a result of either social or environmental changes^15,19–25^. Here, we investigated whether variation in social complexity and brain morphology together can predict cognitive performance in a wild population of cleaner fish *Labroides dimidiatus* (hereafter ‘cleaner’). Previous research on cleaners has shown that variation in social complexity can be well captured within a single parameter, namely, local cleaner density^26^. High densities increase the scope for intraspecific social competition in a protogynous hermaphrodite (where individuals are born as females and change sex to a male later in life), where only the largest individuals turn into males that have access to a harem of females^27^. High densities also lead to an increase in competition over access to ‘client’ fish species that visit cleaners in order to have their ectoparasites removed^28^. When cleaner densities are high, ‘visitor’ species with access to several cleaners are more likely to exert partner choice, i.e. they may switch to another cleaner if made to wait in favour of another client. Therefore, cleaners should give priority to visitor client species over ‘resident’ client species, as individuals of the latter only have access to their local cleaner and must thus wait for service^28^. As local cleaner densities are also highly correlated with visitor client densities, high cleaner densities indicate an overall complex interspecific social environment^26^. Several studies show that cleaners are able to distinguish resident client species from visitor species as they treat them differently^26,28–31^, though the precise mechanisms are not known.

One relevant laboratory-based task that simulates the situation where a resident and a visitor client simultaneously seek cleaning services is called the biological market or ephemeral reward task. It consists of presenting the focal subject with a choice between two Plexiglas plates that both offer an equal food reward, but one is retracted if not chosen first (i.e. the ephemeral or visitor plate). At the same time, the other plate will remain until the subject has eaten the food on it (i.e. the permanent or resident plate). The optimal solution in this task is thus to give priority to the ephemeral option. Overall, cleaner fish perform very well in this task compared to primates^32,33^, rats^34^ and birds^35,36^. Also, cleaners from areas of high population density often outperform cleaners from low-density areas in ecologically relevant tasks^26,37,38^, at least before major environmental perturbations caused severe declines in fish densities^38,39^ As such, cleaners from high densities successfully learned to prioritise the visitor plate (i.e. ephemeral plate) over the resident plate (i.e. permanent plate), whereas cleaners from low densities failed the task^37,38^.

It is beneficial for cleaners to learn the complex task of prioritising the visitor client species over resident client species, but only under certain circumstances^26,40^. As it stands, service priority to visitors should be the best strategy at higher cleaner population densities for several reasons: First, because competition between cleaners over access to visitor clients is higher when the ratio of supply-to-demand is high (i.e. cleaner-to-visitor-client ratios)^41^. Secondly, from the visitor client’s perspective, the option of switching partners is ‘cheap’ when there are many cleaners, making it easy for them to swim to a different cleaner if made to wait for the cleaning service^26,38^. In this context, recent findings showed that major environmental perturbations locally reducing cleaner fish densities by 80% led to a decline in cleaner performance in the biological market task^38^. Such decline was accompanied by a shift in the visitor’s decision-making when made to wait for the cleaning service. When there were fewer cleaners on the reef, visitor clients became more willing to wait for the cleaning service instead of swimming away^38^. This pattern was found across several reef sites where low cleaner density was accompanied by a low switching rate of visitors when made to wait for the service, as well as low cleaner performance in the biological market task, and vice versa at high densities^26^.

In terms of the social competence hypothesis – the ability to optimise social behaviour depending on the available social information^42,43^ – it appears that both high and low performing cleaners can be viewed as socially competent individuals when they are from high and low-density habitats, respectively. Providing service priority to a visitor that might swim away if ignored is optimal as it maximises the chances of a cleaner accessing an ephemeral food source^28^. When visitors are willing to wait, however, it is optimal to ignore the distinction between resident and visitor clients. In this case, cleaners base their service priority, to maximise food intake, on other criteria like the client’s body size^44^ and ectoparasites load^45^. Thus, cleaner decision-rules in the biological market task are likely to be locally adaptive, acquired through experience and learning. First, because cleaner fish have a pelagic larvae stage with little choice over the settlement site^46,47^, which might contribute considerably to gene structure homogenisation^48^. A genetic basis for the observed variation in cleaner decision-rules is hence unlikely. Secondly, cleaners are highly territorial fish^27^, making it unlikely for them to migrate to another reef. And finally, fluctuations in fish population densities can occur between subsequent years^39^, resulting in performance change over the lifetime of individuals^38^ (i.e. lifespan of about five years^49^). Furthermore, it has been shown that cleaner population density correlates positively with forebrain size^50^, which is comprised of the telencephalon and diencephalon and which harbours an evolutionarily conserved network of brain nuclei involved in the regulation of social behaviour, from fish to mammals^51,52^. Together, these observations suggest that there is an ecology–brain–cognition liaison in this species that may explain individual cognitive performance.

By collecting data on the ecology, brain morphology and cognitive performance of cleaner fish, we test whether success and failure in the cognitive task can be predicted by the size or cell counts of specific brain parts while accounting for population density. We find that large forebrains enable individuals to show social competence by performing locally adaptive decision-rules, suggesting forebrain size is important for complex social decision-making.

## Results

To test the ecology–brain–cognition liaison, we first surveyed cleaner fish density at four different reef sites around Lizard Island, in Australia. We then caught and tested a total of 40 female cleaners from these sites to evaluate their performance in the ephemeral reward task. It is important to note that we did not expect the exposure of individuals to the cognitive test to impact brain plasticity per se^53^.

**Fig. 1:**
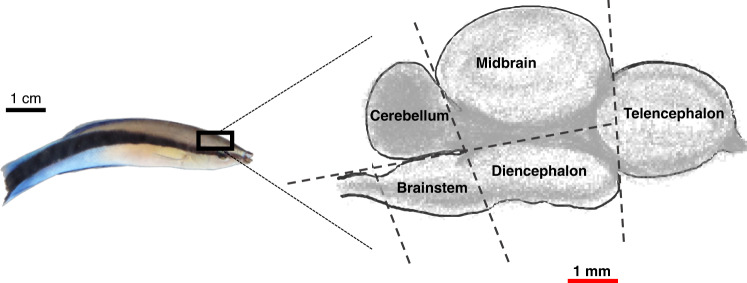
A schematic representation of the cleaner fish (*Labroides dimidiatus*) brain. Cleaner fish brains were dissected into five major brain parts. The telencephalon and diencephalon together form the forebrain. Photo and illustration by Z. Triki.

Only female cleaners were collected for this study since the population is female-biased^27^, and also to facilitate the comparison to previous studies on female cleaner fish^32,37,38,50^. Twenty cleaners were selected for brain analysis based on performance and site of capture: ten individuals that solved the task (high-performers) and ten individuals that failed to solve the task in 200 trials (low performers) from all four sites. Afterwards, we assessed the weight and cell count of five major brain parts: telencephalon, diencephalon, midbrain, cerebellum and brain stem (Fig. 1). We used brain part measurements as proportions of the total brain^2,50^ (see ‘Methods’ section); this is a scaling method designed to identify possible disproportional changes in a particular brain part over the rest of the brain, which helps to identify selective enlargement of specific brain parts^54^.

The twenty adult female cleaners included in the study measured (mean ± SD) 7.43 ± 0.61 cm body total length TL, 3.52 ± 0.88 g body mass, 39.78 ± 7.02 mg brain mass, with 39,701,250 ± 7,515,848 total brain cells (further information in the Supplementary Tables S1 and S2).

**Fig. 2:**
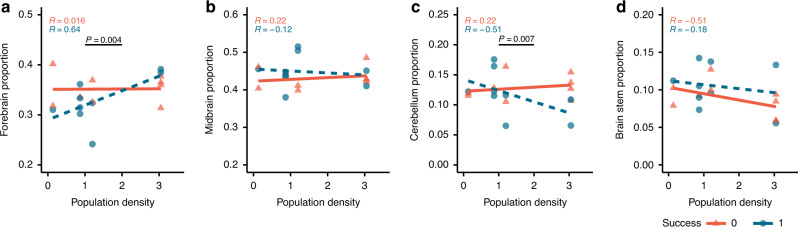
Relationship between performance, brain part sizes, and population density. **a**–**d** Scatterplots and linear regressions of proportions of each brain part size from the total brain size. *p-*values indicate significant (*p* < 0.05) interaction between brain part size and cleaner population density estimated from Generalised Linear Models. *R* coefficient refers to the Pearson correlation coefficient. Red triangles refer to failure while blue circles refer to success in the biological market task. Sample size in **a**–**d** is *n* = 18 biologically independent fish.

We found a significant interaction between relative forebrain size and cleaner population density in predicting performance (Generalised Linear Model (GLM): *X*^2^ = 8.291, *p* = 0.004) (detailed statistics are reported in Table 1). The effect consisted of a positive relationship between relative forebrain size and cleaner population density within the high-performers, while there was no apparent relationship within the low performers (Fig. 2a). From testing the other brain parts, it appears that there was a potential size trade-off between the forebrain and cerebellum, as the latter showed a significant interaction effect with cleaner population density but of an opposite trend than that observed with the forebrain (GLM: *X*^2^ = 7.194, *p* = 0.007, Fig. 2c, Table 1). Neither the midbrain nor the brain stem relative sizes predicted cleaner performance (Fig. 2 and Table 1).

**Table 1:** Relationship between performance, brain neuroanatomical traits and population density.

Fitted model	*N*	Chi^2^	*p*-value	rank (i)	FDR-derived significance threshold	Pseudo-*R*^2^	95% CI		Effect size (Cohen’s *d*)
							Upper	Lower	
Performance ~ Forebrain size proportion * density									
Forebrain size proportion	18	0.563	0.453	20	0.0909	**0.42**	−5.29	2.39	0.05
Density		0.030	0.862	27	0.1227		−1.43	6.99	0.12
Forebrain size proportion × density		**8.291**	**0.004**	1	**0.0045**		−**23.69**	−**1.36**	**0.86**
Performance ~ Midbrain size proportion * density									
Midbrain size proportion	18	1.037	0.309	14	0.0636	–	−2.09	0.62	0.07
Density		0.007	0.933	30	0.1364		−1.64	1.35	0
Midbrain size proportion × density		0.646	0.422	19	0.0864		−0.84	2.53	0.06
Performance ~ Cerebellum size proportion * density									
Cerebellum size proportion	18	1.257	0.262	12	0.0545	**0.41**	0.07	12.62	0.43
Density		0.002	0.968	31	0.1409		−0.42	10.69	0.28
Cerebellum size proportion × density		**7.194**	**0.007**	2	**0.0091**		**1.45**	**31.7**	**0.68**
Performance ~ Brain stem proportion * density									
Brain stem size proportion	18	0.870	0.351	17	0.0773	–	−2.04	0.68	0.09
Density		0.032	0.858	26	0.1182		−1.59	1.24	0
Brain stem size proportion × density		0.001	0.976	32	0.1455		−1.26	1.29	0
Performance ~ Forebrain cell proportion * density									
Forebrain cell proportion	20	0.158	0.691	25	0.1136	–	−1.02	1.66	0.01
Density		0.448	0.503	21	0.0955		−2.29	0.95	0.04
Forebrain cell proportion × density		0.000	0.990	33	0.1500		−1.36	1.36	0
Performance ~ Forebrain cell density * density									
Forebrain cell density	18	0.008	0.930	29	0.1318	–	−1.83	0.86	0.02
Density		0.008	0.927	28	0.1273		−1.38	1.59	0
Forebrain cell density × density		1.173	0.279	13	0.0591		−0.54	2.21	0.11
Performance ~ Cerebellum cell proportion * density									
Cerebellum cell proportion	20	0.216	0.642	23	0.1045	–	−1.15	2.28	0.04
Density		0.203	0.652	24	0.1091		−1.65	1.58	0.01
Cerebellum cell proportion × density		2.179	0.140	6	0.0273		−2.85	0.32	0.19
Performance ~ Cerebellum cell density * density									
Cerebellum cell density	20	2.200	0.138	5	0.0227	**0.43**	−11.57	−0.63	0.43
Density		0.414	0.520	22	0.1000		−0.61	5.39	0.23
Cerebellum cell density × density		**6.885**	**0.009**	3	**0.0136**		−**35.47**	−**1.8**	**0.62**
Social competence ~ Forebrain size proportion * density									
Forebrain size proportion	**18**	**6.118**	**0.013**	4	**0.0182**	**0.34**	**0.71**	**8.73**	**0.77**
Density		2.096	0.148	7	0.0318		−6.67	0.08	0.39
Forebrain size proportion × density		1.517	0.218	10	0.0455		−1.26	7.04	0.2
Social competence ~ Cerebellum size proportion * density									
Cerebellum size proportion	18	0.976	0.323	15	0.0682	–	−2.11	0.69	0.06
Density		0.790	0.374	18	0.0818		−2.59	0.58	0.13
Cerebellum size proportion × density		1.874	0.171	8	0.0364		−4.63	0.4	0.16
Social competence ~ Cerebellum cell density * density									
Cerebellum cell density	20	1.512	0.219	11	0.0500	–	−0.97	1.86	0.03
Density		1.569	0.210	9	0.0409		−2.55	0.31	0.22
Cerebellum cell density × density		0.876	0.349	16	0.0727		−0.63	3.09	0.06

All statistical tests were two-sided binomial tests.

Values in bold indicate statistically significant outcomes. Significance threshold alpha was set at *p* ≤ 0.05. Due to multiple comparisons with a total of eleven models, values were then confirmed with a False Discovery Rate significance threshold adapted to each *p*-value.

False Discovery Rate (FDR)-derived significance threshold was estimated with the following function: (*i*/*m*)*Q*, (with *i*: *p*-value rank; *m*: number of comparisons (here are 11 models); *Q*: maximum acceptable FDR set at 0.05^67^).

Pseudo-*R*^2^ is a goodness-of-fit measure estimated as 1 − (residual deviance/null deviance).

Cohen’s *d* interpretation: 0.2, small; 0.5, medium; ≥0.8 large effect.

*95% CI* 95% confidence interval.

**Fig. 3:**
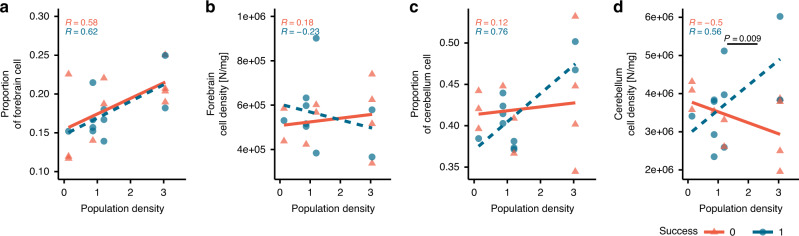
Relationship between performance, forebrain and cerebellum cell count measurements, and population density. Scatterplots of **a** forebrain cell proportion from the total brain cell count, **b** forebrain cell density, **c** cerebellum cell proportion from the total brain cell count, and **d** cerebellum cell density. The *p-*value indicates significant (*p* < 0.05, GLMs) interaction between cerebellum density and cleaner population. *R* coefficient refers to the Pearson correlation coefficient. Red triangles refer to failure while blue circles refer to success in the biological market task. Sample size in **a**, **c** and **d** is *n* = 20 biologically independent animals. In **b**, there is *n* = 18 biologically independent animals.

Because only forebrain and cerebellum sizes showed a significant relationship with population density and cognitive performance, data analyses on cell counts and cell densities (i.e. the number of cells in a brain part divided by the weight of that part) were restricted to these two brain parts. This helped to focus the analyses towards more meaningful questions while reducing the number of multiple comparisons. The cell proportions (i.e. the number of cells in a brain region divided by total brain cells) of neither the forebrain nor the cerebellum predicted performance (Fig. 3). Nevertheless, cerebellum cell density as a function of population density significantly predicted performance (GLM: *X*^2^ = 6.885, *p* = 0.009, Table 1). Here, the high-performers and low performers had, respectively, positive and negative slopes of cerebellum cell density and population density (Fig. 3d).

**Fig. 4:**
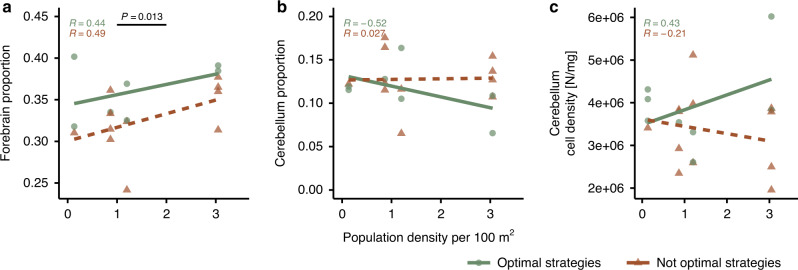
Relationship between locally adaptive behavioural strategies, forebrain and cerebellum measurements, and population density. Scatterplots of **a** forebrain size proportion, **b** cerebellum size proportion, and **c** cerebellum cell densities. Cleaner performance is categorised here as either optimal or not-optimal strategies as a function of population density (see ‘Methods’ section). The *p-*value indicates significant (*p* < 0.05, GLM) differences in forebrain size between individuals with optimal strategies and those with not-optimal strategies. *R* coefficient refers to the Pearson correlation coefficient. Green circles refer to optimal strategies while brown triangles refer to not-optimal strategies within social competence context. Sample size in **a** and **b** is *n* = 18 biologically independent animals. In **c**, there is *n* = 20 biologically independent animals.

In order to understand better the present results (Fig. 2a), instead of using ‘high’ and ‘low’ performance, we relabelled cleaner performance as ‘optimal’ and ‘not-optimal’ according to the social competence hypothesis. To do so, we set a critical threshold of cleaner population density that determines which decision-rule is locally adaptive, which was set at 1.5 cleaner fish per 100 m^2^. We estimated this threshold from the study by Triki et al. (see Fig. 2c in ref. ^26^). Below the threshold, the optimal strategy in nature is to ignore client choice options in favour of other criteria, which then prevents subjects to prioritise the ephemeral food source in the laboratory task. Above the threshold, the optimal strategy is to provide service priority to visitors and thus prioritise the ephemeral food source in the task. With the new labelling, we reran the analyses only for the brain region measurements showing a significant interaction with population density (i.e. forebrain size, cerebellum size, and cerebellum cell density) in predicting performance in the previous analyses. With the new analyses, we tested predictions for social competence instead^42,43,55^. Here, we found that individuals making optimal strategies, that is, having social competence, had relatively larger forebrains than those adopting not-optimal strategies (GLM: *X*^2^ = 6.118, *p* = 0.013, Fig. 4a, Table 1). Cerebellum size and cerebellum cell density did not predict social competence (*p* > 0.05, Fig. 4c, d, Table 1).

## Discussion

Our study showed that linking social complexity, brain features and cognitive performance in wild animals is rather complex. By incorporating information about the ecology of cleaning interactions as a function of cleaner fish densities, the concept of social competence emerged as a suitable framework to link forebrain size to cognitive performance (see review by Varela et al.^55^). As it stands, ecological conditions can determine what decision-rules are locally adaptive and apparently a larger forebrain facilitates the acquisition of these rules. As long as wild-caught subjects use their previous experiences and learned decision-rules in laboratory-based cognitive tasks that tap into these experiences, and if experiences vary across subjects, performance becomes a poor predictor of intelligence. With 20 test trials a day for ten days, we were still far from the natural frequency of a visitor and resident client simultaneously seeking cleaning service, which ranges between 2.8 and 38.6 occasions per 30 min (~62 and 850 occasions a day—assuming 11 h of cleaning activity per day), according to the behavioural observations of 112 cleaner fish from variable population densities around Lizard Island^26^. Indeed, our task pre-defined that a sophisticated decision-rule yields the highest food reward, which probably created a mismatch between what is optimal in the laboratory task and what is optimal in nature for individuals from low population density sites. Therefore, leading to highly socially competent (i.e. more intelligent) fish possibly performing poorly in the task. Only knowledge of each individual’s social environment allows the use of social competence as an alternative approach to evaluate an individual’s ‘intelligence’ in wild populations whenever experiments tap into previous experience. Our findings are in line with Thornton and Lukas’ predictions that rearing conditions and previous experience can have tremendous impacts on the measured cognitive performance^14,24^. Resulting individual differences are usually considered the noise around the population mean, but our study suggests that they can be explicitly linked to key factors like ecological conditions and brain morphology. Thus, we extrapolate from our fish study to other vertebrate species that not all individuals can show adaptive plasticity to local conditions, but only those with enough brain capacity. However, the need to adapt to local conditions might be the driver that generates enough brain capacity.

Importantly, these findings show that forebrain cell counts do not predict cleaners’ performance in the ephemeral food task, neither directly nor as a function of population density. The fact that forebrains of socially competent individuals were larger without an increase in cell numbers, suggests a neuropil volume expansion, instead^56,57^. Unfortunately, the information on neuron numbers was missing (see ‘Methods’ section), making it difficult to estimate the variation in the proportion of neurons to glia. If one assumes that the number of glial cells remained constant, then the potential increase in neuropil volume must be due to a higher volume of dendrites and axons, concomitant with increased neural connectivity^58^. Thus, an enlarged forebrain might reflect higher connectivity rather than an increase in the number of brain cells.

Given that our experimental design explicitly selected high and low performers from different densities (i.e. the 20 female cleaners for brain analyses), there could not be the main effect of cleaner density on performance in this sub-sample (see Methods). Furthermore, forebrain cell numbers did not predict performance in either task, but they positively correlated with cleaner population density. For instance, the cell proportion in the forebrain was 40% higher in cleaners from the reef site with the highest cleaner population density compared to the sites with the poorest population density (Fig. 3a). This fits previous findings documenting that cleaners from high population density sites have larger forebrains than cleaners from low-density populations^50^. Thus, the factors that are causing such relationships between population density with either forebrain size or forebrain cell counts are not well captured by the biological market task. As this experiment captures key aspects of cleaners’ interspecific social interactions, an avenue for future research is to explore the links between brain features and social competence at the intraspecific level. In addition, other factors like environmental enrichment^15,25,53^ should be considered for further studies to understand better brain morphology in cleaner fish.

The apparent trade-off between forebrain and cerebellum sizes (Fig. 2) is interesting, given that we are not aware of any literature that yields predictions regarding such a trade-off. However, our findings suggest that there is an effect. The forebrain and the cerebellum have been classically seen as performing qualitatively different functions. Whereas the telencephalon has been viewed as an executive centre for higher cognitive processing, the cerebellum has been seen as having a role in the balance of the body and motor coordination, due to its afferent connections with the vestibular and somatosensory systems^59^. However, in the last decades, evidence has accumulated showing that the cerebellum can also have an important role in cognitive functions, namely in the precise timing and detection of temporal relations of events^58^. Irrespective of the functional role of this apparent trade-off, it leads to a compression of the cerebellum, causing an increased cell density without changing absolute cell numbers (Supplementary Fig. S1). The fact that across different animal taxa the cerebellum contains the largest numbers of neurons that are typically highly packed^60^ suggests that this brain region is prone to compression, possibly due to the smaller size of some of its cells (e.g. granule cells in the cellular layer). Whether or not such a compression lowers the functionality of the cerebellum (see review by Niven and Farris^61^), thereby leading to a functional trade-off with increased forebrain size, is currently unclear.

In conclusion, our study suggests a causal link between forebrain size and social competence that allows individuals to adjust to local ecological conditions. Bridging development and evolution will be necessary to understand to what extent cleaner fish are currently under selection to express high levels of social competence.

## Methods

### Field site and fish survey

The study was conducted at four different sites at Lizard Island (14.6682° S, 145.4604° E), Great Barrier Reef, Australia, between July and August 2018. To estimate cleaner fish population densities, scuba divers conducted an underwater fish survey at each study site. Observers counted cleaner fish abundance on transect lines. In total, a replicate of ten transects of 30 m each was conducted at every study site, except at The Crest (14° 41′37.9″S 145° 27′58.3″E), where seven replicates were collected. The transect line was either placed parallel to the reef crest (i.e. at Mermaid cove, −14.647792, 145.454106; and The Crest) or parallel to the shoreline (i.e. at Northern horseshoe, −14.685167, 145.443293; and Corner beach, −14.673398, 145.440601). On every transect line, observers recorded the number of adult cleaners within a 5 m width (i.e. 2.5 m on either side of the transect line). Cleaner counts were then scaled to densities per 100 m^2^.

### The biological market task (Ephemeral reward task)

For laboratory experiments, 40 adult female cleaner wrasse *Labroides dimidiatus* (total length TL: mean ± SD, 7.43 ± 0.61 cm) were collected from the four study sites (i.e. The Crest, Mermaid Cove, Northern Horseshoe, and Corner Beach). Scuba divers captured cleaners with barrier nets (2 m × 1 m, 5 mm mesh size) and hand nets. At Lizard Island Research Station facilities, all fish were individually housed in glass aquaria (62 cm × 27 cm × 37 cm) and provided with PVC pipes (10 cm × 1 cm) as shelters. All fish were allowed an acclimation period of at least 14 days before proceeding with the laboratory experiments. Fish were fed daily with a paste of mashed prawn smeared on Plexiglas plates (8 × 15 cm). During Laboratory experiments, cleaners received food from the trials from 8:00 to 17:00. Before the ephemeral reward task, all cleaners had been tested in an ‘audience effect’ task. Unfortunately, this task did not generate the desired effect of having enough variation to classify individuals as high- or low-performers as they all performed poorly in this task (Supplementary Methods).

In the biological market task, we tested cleaners for their abilities to learn to prefer a visitor plate (ephemeral food source) over a resident plate (permanent food source). We used Plexiglas plates as surrogates for client fish. The plates were of equal size (10 cm × 7 cm) and offered an equal amount of food (i.e. one prawn item each). To facilitate visual discrimination of the two plates, we ensured that both plates had either vertical pink stripes or horizontal green stripes as decoration^26,37,38^. The plate with a resident role was always willing to stay in the aquarium until the cleaner fed on it. The visitor plate, however, was an ephemeral food source that was accessible only if it was given priority of inspection by the cleaner. Otherwise, the visitor plate would be withdrawn from the aquarium if the cleaner inspects the resident plate first. For each trial, cleaners would be confined to one side of the aquarium by two separations (i.e. one opaque and one transparent) while the two test plates were being placed on the other side of the aquarium. We first removed the opaque barrier, then the transparent one allowing the fish to see the experimental set up for a few seconds before it was released. This helped to avoid impulsive choices due to the speed of swimming and reaching for the closest Plexiglas plate. For ten consecutive days, we ran a total of 200 trials per fish. We tested every fish in two sessions (i.e. one session constituted ten trials) a day, with one session in the morning and one in the afternoon. To solve the task, a cleaner had to show a significant preference towards the visitor plate, which consisted of a score of either: nine or more successful choices out of a session of 10 trials; two consecutive eight successful choices out of sessions of 10 trials each; three consecutive seven successful choices out of sessions of 10 trials each. Also, the decoration (i.e. vertical pink stripes or horizontal green stripes) and the status of the plates (i.e. visitor or resident) were counterbalanced between fish. At the same time, the spatial location (i.e. left or right) was randomised and counterbalanced between trials. That is, there were no more than three trials in a row for the same spatial location, with a 50:50 ratio for a plate to be presented on either side.

### Study animals and brain sampling

Testing 40 adult female cleaners in a complex foraging task like the biological market task (ephemeral reward task) over 200 trials allowed us to evaluate their cognitive performance in an ecologically relevant task. From there, we selected ten high-performers and the ten low-performers for the brain analyses (Supplementary Table S1). The ten high-performers all successfully solved the biological market, while the ten low-performers all failed the task. Since cleaners have a key role in coral reef fish health^62,63^, diversity and abundance^64^, we limited brain collection to 20 individuals, while the other 20 females were returned to their home reef at the end of data collection. Also, the field site at Lizard Island recently suffered from a severe decline in fish densities following consecutive environmental perturbations, like cyclones and coral bleaching^38,39,65^. The directors of the Lizard Island Research Station, Dr Anne Hogget and Dr Lyle Vail are concentrating efforts to sustain coral reef and fish communities recovery. After consulting with them regarding this project, it was agreed to sample 20 fish instead of 40, and we thus returned the other 20 individuals to their home reef to reduce disturbances due to absence of cleaners on coral reefs^62,64^.

Selected cleaners were sacrificed by a rapid cervical transection. Immediately after, the upper part of the skull was removed to allow easy access to the brain tissue. We dissected the brain into five main brain parts under a stereomicroscope (Zeiss steREO Discovery.V8) with a zoom set at 7:1. The five brain parts were: telencephalon, diencephalon, midbrain, cerebellum and brain stem, as shown in Fig. 1. Brain tissue was then weighed with an analytical balance with a readability of up to 0.0001 g. The samples were then fixed for 24 h in 4% paraformaldehyde (PFA) solution at 4 °C. The brain tissue was then transferred to PBS 0.1% sodium azide solution and stored at 4 °C. Three brain part samples that belong to two different fish had an error while reading tissue weight, and it was not possible to weigh them again as they were already being transferred into the PFA solution. Nevertheless, we were able to estimate cell counts from these three samples. Samples were shipped from Lizard Island to the Gulbenkian Institute in Lisbon for further brain tissue analyses.

### Brain cells quantification

To estimate total cells and neuron numbers from the dissected brain parts, we followed the isotropic fractioned method^66^. The method consisted of dissociating brain tissue using a tissue grinder. To facilitate dissolving brain cell membranes while simultaneously ensuring that nuclear membranes remain intact, we ground the tissue in a saline detergent solution. Upon tissue homogenisation, samples were stained with diamino-phenyl-indol (DAPI). In a first step, cell counts were performed for every brain part with a haemocytometer (Brand® counting chamber Blaubrand® Neubauer improved) under a microscope with fluorescence (Leica DMRA2) using a ×40 dry lens (0.75 numerical aperture). The second step should have been to run an immunocytochemical protocol to identify the neurons using a neuronal protein marker (anti-NeuN rabbit Antibody, ABN78, dilution 1:100; Merck). However, due to fungal contamination of the samples during storage, it was not possible to run the second step of the technique to estimate neuronal numbers, and we only obtained reliable numbers on total cell numbers. All cell counts were done blindly regarding the identity of the samples.

### Scaling method for brain measurements

We used two scaling methods for brain part weights, as well as for cell counts per brain part: (i) we extracted the residuals from the regression of the log-transformed size of the brain part of the interest on body length. Such scaling methods control for the brain portion responsible for maintenance and allow to compare the brain component supposedly linked to cognitive processing^1^. (ii) We estimated the size proportion of the brain part of the interest from the total brain.

Nevertheless, using both scaling methods for data analyses with a relatively small sample size increased the risk of Type I error, that is, the probability of falsely rejecting the null hypothesis. We decided then to see whether residuals and proportions correlate positively. This helped in keeping one set of the measurements as it represented both scaling approaches (i.e. residuals or proportions). We created a correlation matrix with Pearson’s coefficients that showed proportions correlated with residual measurements most of the time (*r* ≥ 0.8, Supplementary Fig. S1). In other words, an increase in a brain region size while accounting for body size was also accompanied by an increase in that region’s proportion of the whole brain.

In line with our aims, we decided to compute the telencephalon and diencephalon jointly as one unit (i.e. forebrain). The rationale of this decision lays in previous findings by Triki et al.^50^, that shows that the telencephalon and diencephalon together form a key brain part susceptible to changes in sociality levels of cleaner fish. Nevertheless, we still explored potential trade-offs between the telencephalon and diencephalon as a function of cleaner density in the present study. We found that cleaner density had neither significant effect on the size ratio of telencephalon to the diencephalon, nor in their cell count ratio (LMs: *N* = 18, *F*_(1, 16)_ = 0.002, *p* = 0.962; *F*_(1, 18)_ = 0.147, *p* = 0.706, respectively).

### Statistical analyses

All statistical analyses and figures were generated with the open-source software R version 3.6.2. While analytical statistics were used to test our hypotheses, descriptive statistics were used to provide more information about the different measurements collected in the present study. Therefore, supplementary figures were generated to show graphically that there was no systematic co-variation in body size as a function of population density (Supplementary Fig. S3). Other supplementary figures depict the relationship between brain part sizes, cell counts, cell densities and body size (supplementary Figs. S4–S7). Also, a summary table of descriptive statistics for each brain measurement is reported in Supplementary Table S2.

This study aimed to test whether cleaner performance in the biological market task can be predicted by cleaner population density and/or brain part measurements. To do so, we fitted a set of Generalised Linear Models (GLM) with the performance as a binary response variable (i.e. success and failure). Meanwhile, cleaner density and brain part measurement were fitted as continuous predictors. For all the fitted models, we tried to avoid issues with model convergence and inflation of standard errors by centring and standardising all numerical predictors beforehand. That is, values in a numerical variable were subtracted from the mean (i.e. centring), so the new mean is 0, then divided by the standard deviation (i.e. standardisation). This was performed with the built-in function in R language scale(). Statistical outcomes reported in Table 1 were generated by the Anova () function from ‘car’ package in R language that runs Type II sum of the square test. We also checked for models’ assumptions, such as overdispersion. Given the multiple testing of cleaner performance in several statistical models, we employed the False Discovery Rate (FDR) approach to set a significance threshold adapted to every *p*-value^67^. The FDR-derived significance threshold was estimated with the following function: (*i*/*m*)*Q* with *i*: *p*-value rank; *m*: number of comparisons (there are 11 models); *Q*: maximum acceptable FDR set at alpha = 0.05.

Furthermore, the effect size (Cohen’s *d*) was estimated from the partial coefficient of determination (i.e. partial *R*^2^) using the R packages ‘rsq’ and ‘effectsize’ in R language. Post hoc visualisation of the interaction effects between continuous predictors was depicted by the function *visreg2d* from the ‘visreg’ package in R language (Supplementary Fig. S2). A step-by-step statistical code allowing the reproducibility of the present statistics is archived along with the data files.

### Ethical note

The Animal Ethics Committee of the Queensland government (DAFF) approved the project under the permit number CA 2017-05-1063.

### Reporting summary

Further information on research design is available in the Nature Research Reporting Summary linked to this article.

## Supplementary information

Supplementary Information

Peer Review File

Reporting Summary

## Data Availability

Source data are provided with this paper and they are archived at Figshare data repository by Triki et al.^68^ (10.6084/m9.figshare.7415576). Source data are provided with this paper.

## Source data

Source Data

## Code availability

Analyses reported in this article can be reproduced using code provided by Triki et al.^68^ (10.6084/m9.figshare.7415576).
