# Supplementary material for: Brain morphology predicts social intelligence in wild cleaner fish
Source: Nat Commun. 2020 Dec 21;11:6423. doi: 10.1038/s41467-020-20130-2 (PMC7752907; doi:10.1038/s41467-020-20130-2)
Supplement: Supplementary file 1 — Supplementary Information [file 41467_2020_20130_MOESM1_ESM.pdf]

Supplementary Material for: Brain morphology predicts social intelligence in wild cleaner fish

Authors: Zegni Triki[1,2*], Yasmin Emery[1], Magda C. Teles[3] , Rui F. Oliveira[3,4] , Redouan Bshary[1]

Affiliation:

[1]Institute of Biology, University of Neuchâtel, Emile-Argand 11, 2000 Neuchâtel, Switzerland

[2]Department of Zoology, Stockholm University, Svante Arrheniusväg 18 B, Stockholm, Sweden

[3]Instituto Gulbenkian de Ciência, Rua da Quinta Grande, 6, 2780-156 Oeiras, Portugal

[4]ISPA – Instituto Universitário, Rua Jardim do Tabaco 34, 1149-041 Lisboa, Portugal

***Correspondence to:** Zegni Triki, email: zegni.triki@gmail.com

Supplementary Methods:

In this study, the 40 adult female cleaners were also tested in another task called the "audience effect" task. Unfortunately, this task did not generate the desired effect of having enough variation to classify individuals as high- or low-performers as they all performed poorly in this task. Therefore, it was not possible to test our hypothesis with such data. The dataset from all 40 cleaners can be accessed through the link in the data accessibility statement. However, here we add the data from the 20 cleaners selected for the brain analyses as a supplementary Table S2.

In this experiment, fish were presented with two types of food: one is highly preferred food, and the other is less preferred. From previous research, we know that cleaners prefer eating prawn (i.e., highly preferred food) over fish flakes (i.e., less preferred food) in a choice test[1]. Therefore, we used prawn and flake food items to mimic client mucus and ectoparasites, respectively. To ensure that fish distinguish between these two types of foods, we subjected them to a training phase before testing them in the audience effect task. In this phase, cleaners were trained to first eat less preferred food items (i.e., flakes) before eating a highly preferred food (i.e., prawn). In total, we ran five learning trials over two days. In every single trial, a Plexiglas plate offering 12 flake items and two prawn items was introduced to the fish. Cleaners were permitted to eat flake items. Consuming a prawn item, however, resulted in the withdrawal of the plate from the aquarium. The same plate with the remaining food items was reintroduced in the aquarium after 60 seconds. Again, the consumption of a prawn item led to the withdrawal of the plate. Once no prawn item remained on the plate, the trial would end. During this training period, all fish experienced eating a flake item before eating a prawn item at least once.

In the test trials, fish were presented with two new Plexiglas plates of similar size (12 cm x 7 cm) exhibiting different decoration of either yellow or white stripes. At this stage, every plate offered four food items in total: two flakes and two prawns. Similarly to the training phase, the plates would remain in the aquarium as long as the fish ate flake items only. Upon eating a prawn item, the plates would leave the aquarium. To maximize food intake, the optimal strategy is to feed on all available flake items before eating one prawn item. The audience effect task had two conditions: treatment and control. In the treatment, whichever plate the cleaners feed from first, makes the second plate a bystander plate. In other words, cleaners can have access to the bystander as long as they eat only flakes off the first plate. It aimed to reproduce natural conditions, where cleaners can have access to an image-scoring bystander client only if they are cooperative with the current client[2]. In the control condition, however, cleaners were presented with a single plate.

The task was composed of several rounds of two trials each, one trial as control and one as treatment. An interval of 30 min was allowed between every two trials, and subsequently a 60 min time interval between two rounds. Randomization of the order of the two trials within each round for every cleaner was determined by flipping a coin. In total, cleaners were subjected to nine rounds, over a period of two days. From here, we summed the number of flakes and also that of prawns consumed during the nine trials for the treatment and control conditions. Then, we calculated the ratio of flakes to prawns consumed for each condition. First of all, cleaners rarely succeed in accessing the bystander plate, as they cheat by consuming a prawn item on the first plate. Second, on average they failed to eat at least one flake item before a prawn in either condition. A possible explanation is that the quality of fish flakes used in the experiment

was not appealing for cleaners in contrast with other brands used before for similar experiment, wherein the ratio of flakes to prawn consumed by cleaners was way higher than our ratios[2].

Supplementary Figures:

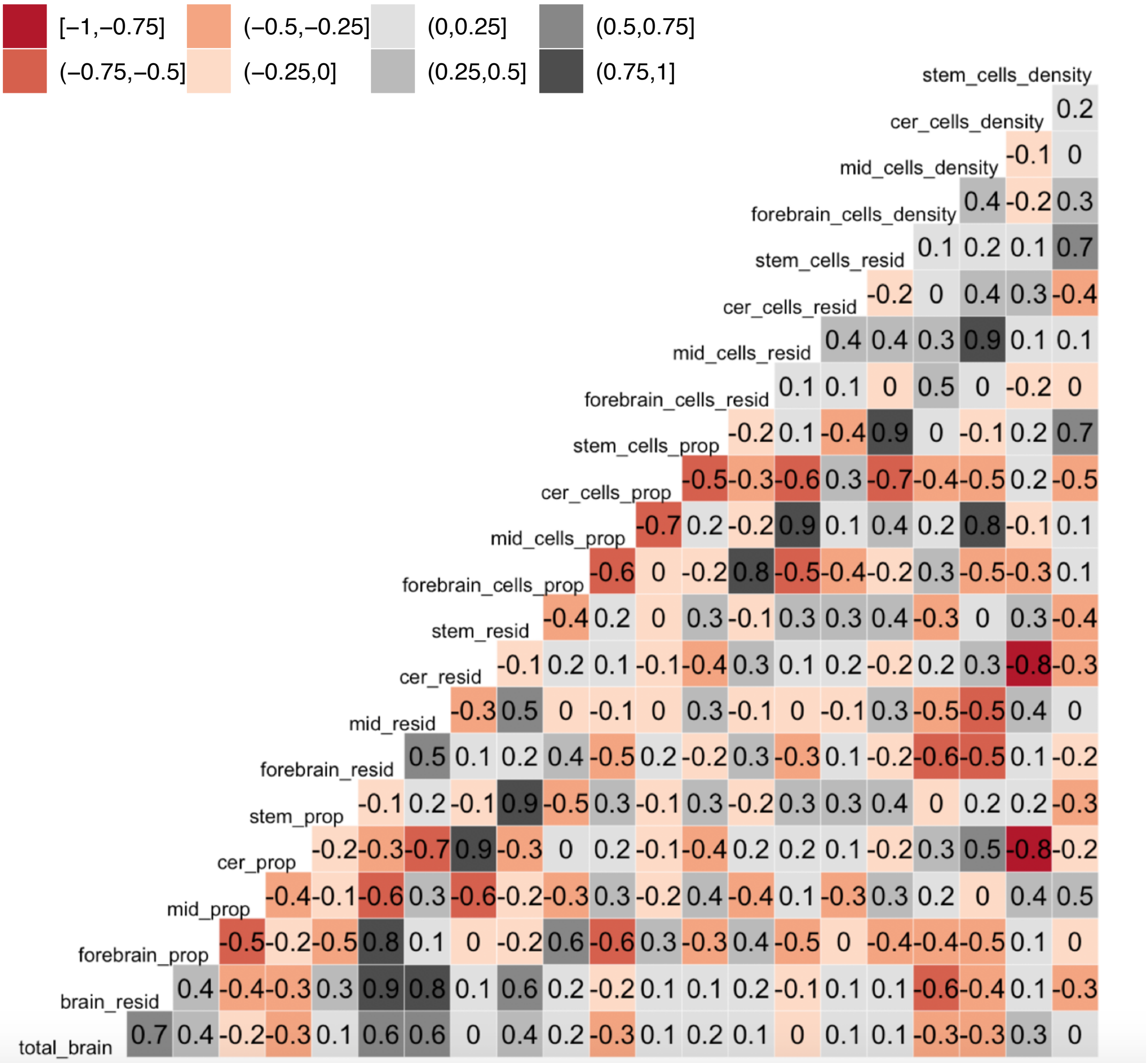

Figure S1. Correlation matrix of all the brain measurements. Depicted are Pearson's correlation coefficients. Abbreviations are: total_brain: brain weight in mg; brain_resid: residuals of the regression of the whole brain size on body length; forebrain_prop: size proportion of the forebrain from the whole brain; mid_prop: size proportion of the midbrain from the whole brain; cer_prop: size proportion of the cerbellum from the whole brain; stem_prop: size proportion of the brain stem from the whole brain; forebrain_resid: residuals of the regression of the forebrain size on body length; mid_resid: residuals of the regression of the midbrain size on body length; cer_resid: residuals of the regression of the cerebellum size on body length; stem_resid: residuals of the regression of the brain stem size on body length; forebrain_cells_prop: cell proportion of the forebrain from the whole brain; mid_ cells_prop: cell proportion of the midbrain from the whole brain; cer_ cells_prop: cell proportion of the cerbellum from the whole brain; stem_ cells_prop: cell proportion of the brain stem from the whole brain; forebrain_ cells_resid: residuals of the regression of the forebrain cell count on body length; mid_ cells_resid: residuals of the regression of the midbrain cell count on body

length; cer_ cells_resid: residuals of the regression of the cerebellum cell count on body length; stem_ cells_resid: residuals of the regression of the brain stem cell count on body length; forebrain_ cells_density: cell density in the forebrain; mid_ cells_ density: cell density in the midbrain; cer_ cells_ density: cell density in the cerebellum; and stem_ cells_ density: cell density in the brain stem.

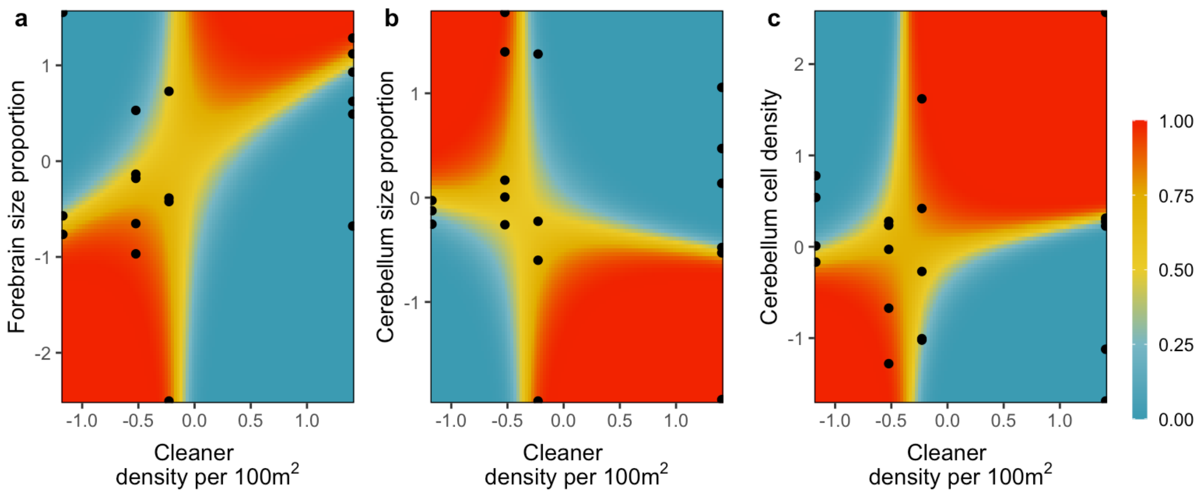

Figure S2. filled contour plot of the significant interaction between brain part measurements and population density. The colour map indicates the model predictions of probability of success in the biological market task where the associated values are colour coded on the right bar. Vertical and horizontal axes are the standardized explanatory variables. The raw data points are depicted in black dots. (**a**), (**b**) and (**c**) are effect display of the significant interactions of two quantitative predictors that are simultaneously varying. Plots generated with visreg package in R language. Sample size in (**a**) is n = 18 biologically independent animals, while in (**b**) and (**c**) there is n = 20 biologically independent animals. P-values for the significant interactions effects were: (**a**) p = 0.004, (**b**) p = 0.007, and (**c**) p = 0.009.

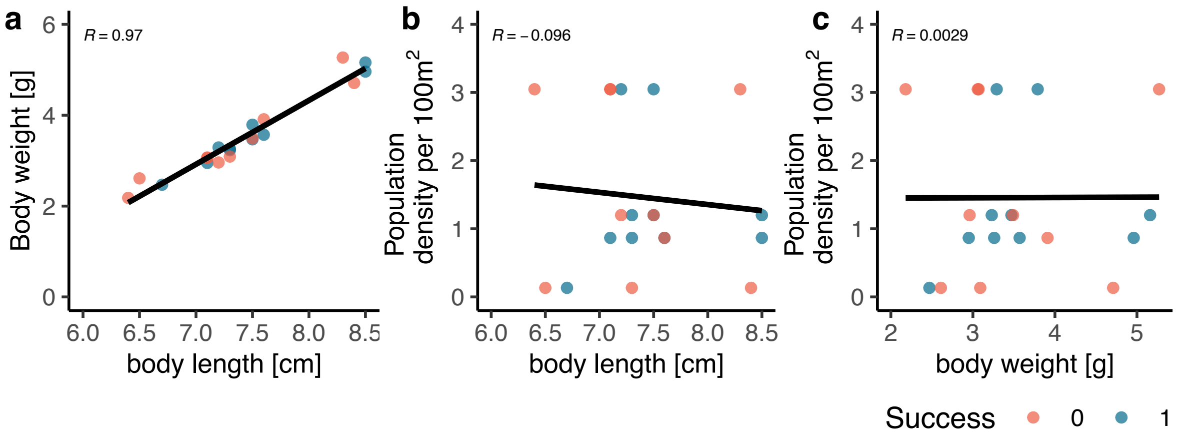

Figure S3. Body size and population density. Scatterplots of the relationship between body length and body weight, and the relationship of these measurements and cleaner population density. *R* coefficient refers to the Pearson correlation coefficient. Red circles refer to failure while blue circles refer to success in the biological market task. Sample size in (**a**) to (**c**) is n = 20 biologically independent animals.

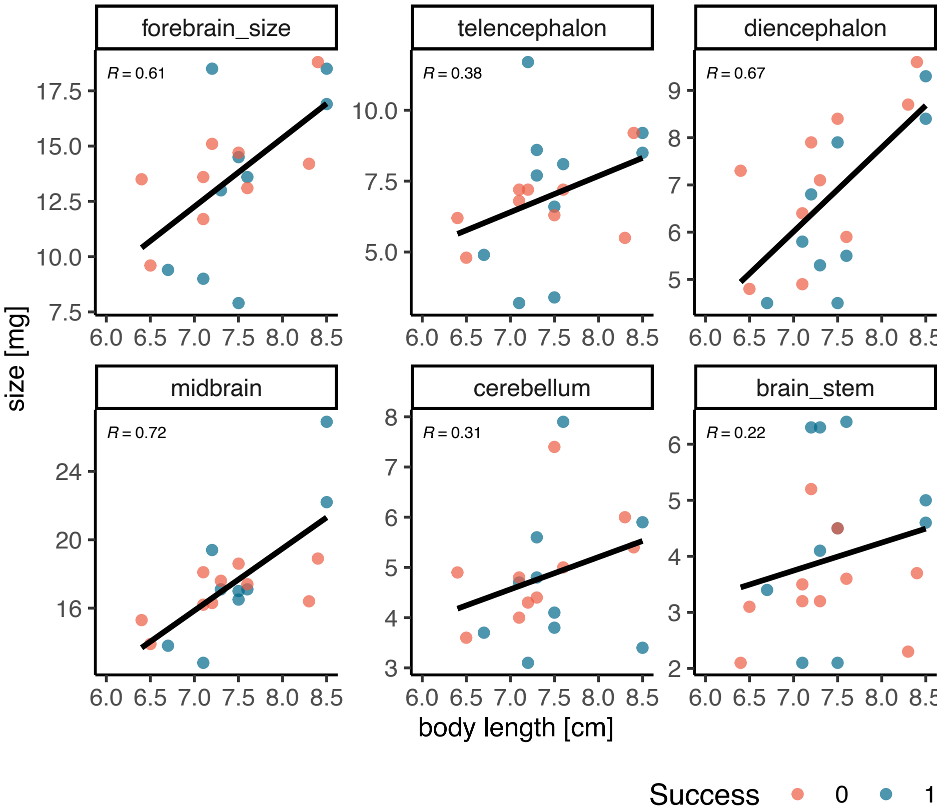

Figure S4. Relationship of brain part absolute sizes and body length. The telencephalon and diencephalon together form the forebrain. *R* coefficient refers to the Pearson correlation coefficient. Red circles refer to failure while blue circles refer to success in the biological market task. Sample size is n = 18 biologically independent animals for forebrain, n = 19 for telencephalon, n = 19 for diencephalon, n= 19 for midbrain, and n= 20 for cerebellum and n= 20 for brain stem.

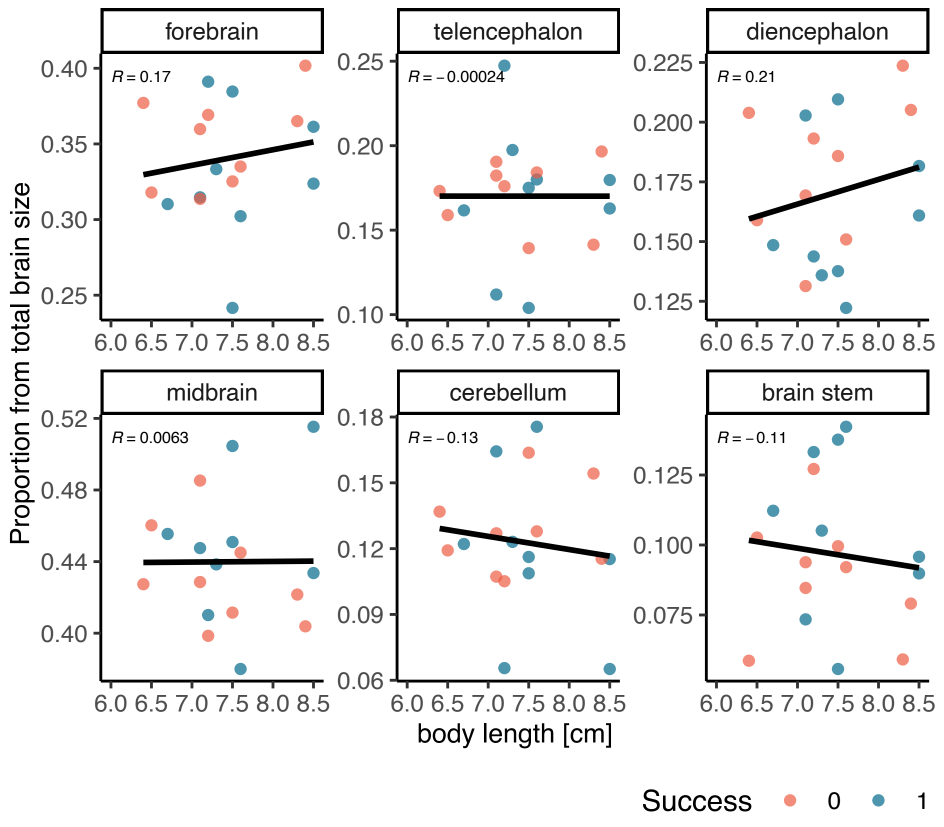

Figure S5. Relationship of brain part size proportions and body length. The telencephalon and diencephalon together form the forebrain. *R* coefficient refers to the Pearson correlation coefficient. Red circles refer to failure while blue circles refer to success in the biological market task. Sample size is n = 18 biologically independent animals in all panels.

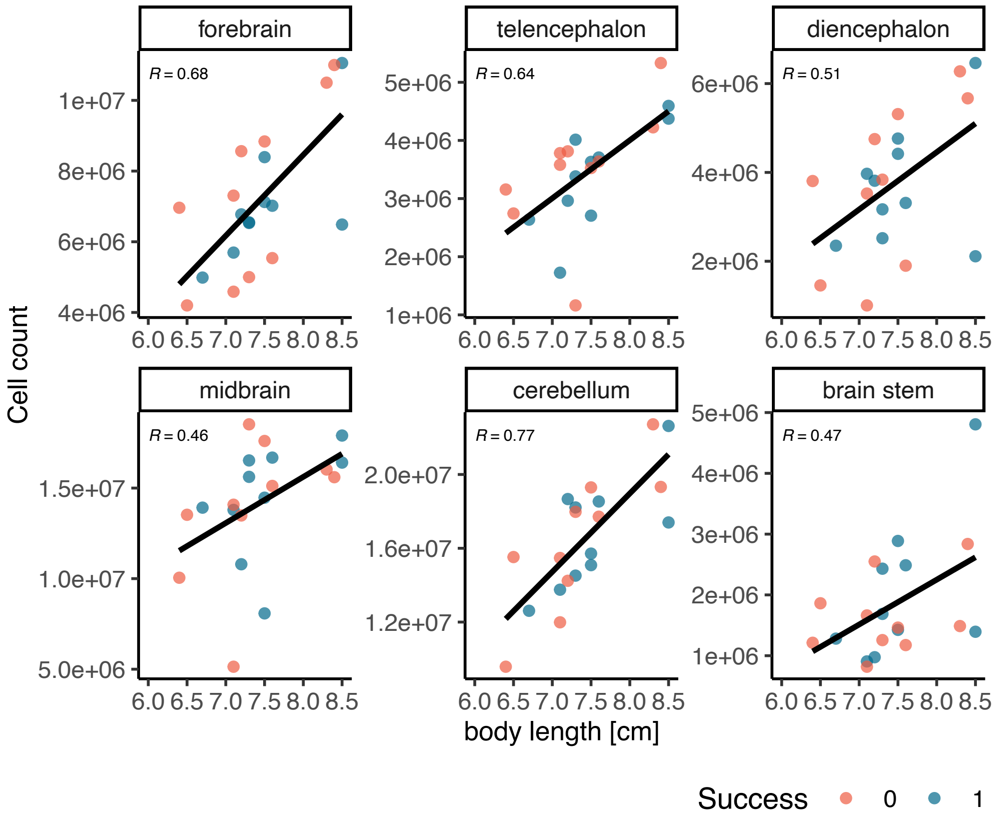

Figure S6. Relationship of brain part cell count and body length. The telencephalon and diencephalon together form the forebrain. *R* coefficient refers to the Pearson correlation coefficient. Red circles refer to failure while blue circles refer to success in the biological market task. Sample size is n = 20 biologically independent animals in all panels.

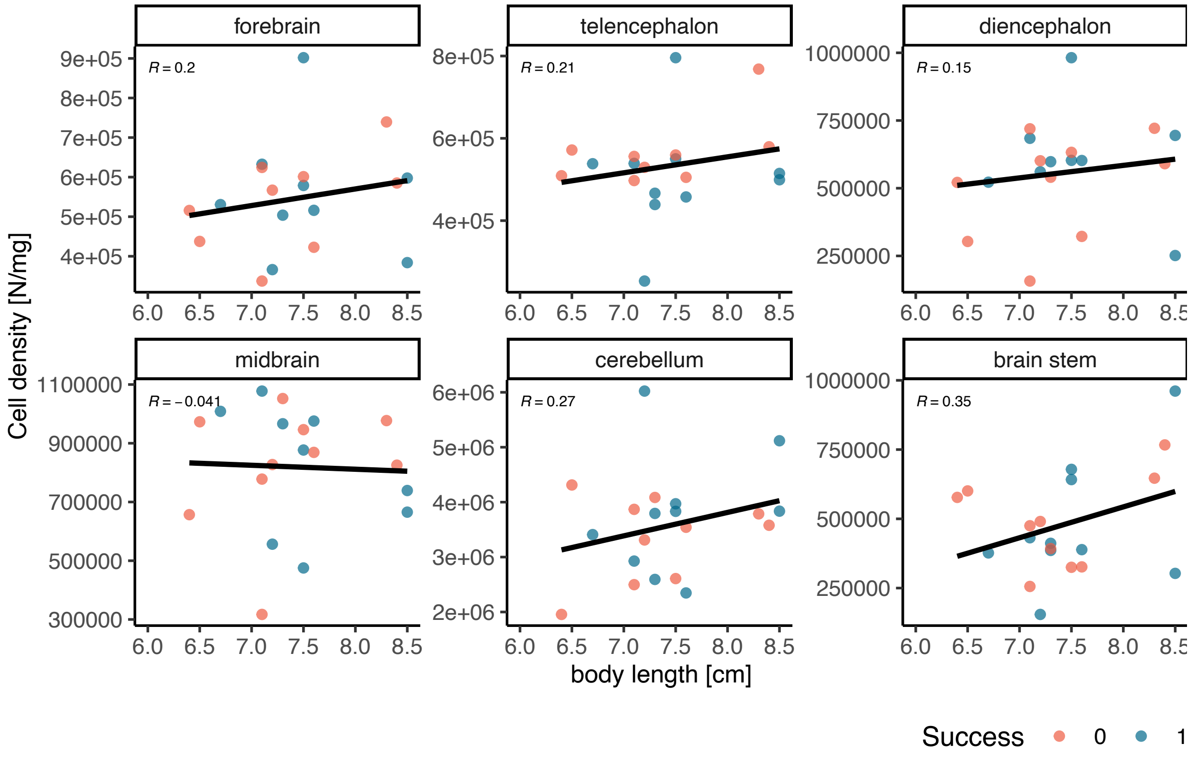

Figure S7. Relationship of brain part cell density and body length. The telencephalon and diencephalon together form the forebrain. *R* coefficient refers to the Pearson correlation coefficient. Red circles refer to failure while blue circles refer to success in the biological market task. Sample size is n = 18 biologically independent animals for forebrain, n = 19 for telencephalon, n = 19 for diencephalon, n= 19 for midbrain, and n= 20 for cerebellum and n= 20 for brain stem.

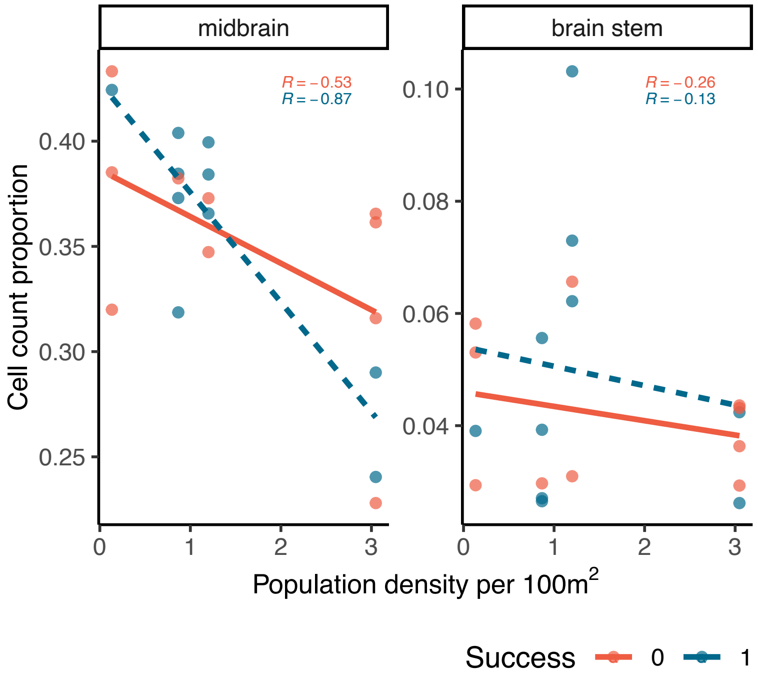

Figure S8. Relationship of brain part cell count and cleaner population density. *R* coefficient refers to the Pearson correlation coefficient. Red circles refer to failure while blue circles refer to success in the biological market task. Sample size is n = 20 biologically independent animals.

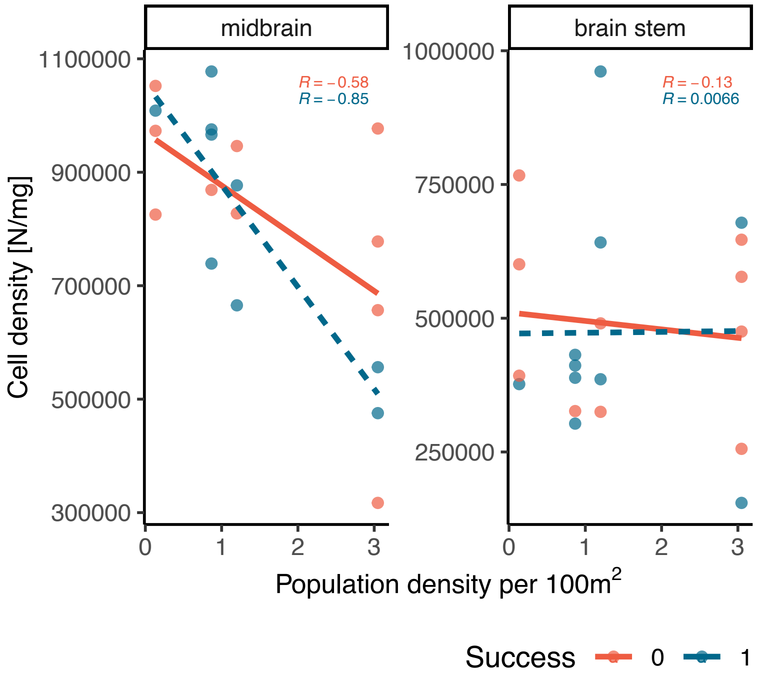

Figure S9. Relationship of brain part cell density and cleaner population density. *R* coefficient refers to the Pearson correlation coefficient. Red circles refer to failure while blue circles refer to success in the biological market task. Sample size is n = 20 biologically independent animals.

Supplementary Tables:

Table S1. Summary table of the sampled fish for brain analyses. The table shows the number of cleaners sampled per reef site and cleaner density. The numbers are shown based on performance in the biological market task (see Methods). The average cleaner density for high- and low-performers is 3.96 and 3.32 (cleaner per 100 m^2), respectively.

reef site	cleaner density per 100 m^2	failure	success
Corner beach	0.133333333	3	1
Mermaid cove	0.866666667	1	4
Northern Horseshoe	1.2	2	3
The Crest	3.047619048	4	2

Table S2. Summary table of the neuroanatomical traits measured in this study. For cell counts, every brain part was reduced to a suspension of cells in 1 mL of PBS solution (see Methods).

Variable	N	Mean	Standard Deviation	Median	Standard Error	Minimum	Maximum
Body length (cm)	20	7.4	0.61	7.30	0.14	6.40	8.50
Body weight (g)	20	3.5	0.88	3.28	0.20	2.18	5.27
Telencephalon size (mg)	19	7.0	2.09	7.20	0.48	3.20	11.70
Diencephalon size (mg)	19	6.8	1.66	6.80	0.38	4.50	9.60
Midbrain size (mg)	19	17.4	3.14	17.10	0.72	12.80	26.90
Cerebellum size (mg)	20	4.8	1.26	4.75	0.28	3.10	7.90
Brainstem size (mg)	20	4.0	1.38	3.65	0.31	2.10	6.40
Total brain size (mg)	18	39.8	7.02	38.95	1.65	28.60	52.20
Telencephalon cell count per 1ml	20	3434375.00	953159.78	3606250.00	213133.01	1162500.00	5331250.00
Diencephalon cell count per 1 ml	20	3721250.00	1544496.81	3809375.00	345359.99	1006250.00	6462500.00
Midbrain cell count per 1ml	20	14166562.50	3388396.46	14793750.00	757668.48	5137500.00	18518750.00
Cerebellum cell count per 1ml	20	16548750.00	3346757.19	16556250.00	748357.66	9581250.00	22718750.00
Brainstem cell count 1 ml	20	1830312.50	946110.19	1475000.00	211556.67	818750.00	4806250.00
Total brain cell count	20	39701250.00	7515848.00	39321875.00	1680594.70	22531250.00	51487500.00

Table S3. Data table of the cleaner fish performance in the audience effect task. The scores for the control plate and the bystander plate were estimated by dividing the number of flakes by the number of prawns consumed by cleaners on each plate. First refers to the first plate of the two plates presented to cleaner fish in the treatment condition: whichever plate is serviced first was labelled as “first”. It shows the number of items eaten by cleaners on that plate in the presence of a bystander plate.

cleaner_ID	control_flake	control_prawn	first_flake	first_prawn	control_score	first_score	cleaner_density
CL 03	9	9	7	8	1.00	0.88	1.20
CL 11	6	9	2	9	0.67	0.22	0.13
CL 24	12	9	12	9	1.33	1.33	0.87
CL 27	4	9	6	8	0.44	0.75	0.87
CL 36	6	9	7	9	0.67	0.78	3.05
CL 38	6	9	5	9	0.67	0.56	3.05
CL 01	7	9	11	7	0.78	1.57	1.20
CL 02	10	9	8	7	1.11	1.14	1.20
CL 28	5	9	8	6	0.56	1.33	0.87
CL 29	4	9	9	7	0.44	1.29	0.87
CL 04	9	9	7	8	1.00	0.88	1.20
CL 05	9	9	7	9	1.00	0.78	1.20
CL 15	8	9	5	9	0.89	0.56	0.13
CL 17	4	9	3	8	0.40	0.38	0.13
CL 18	7	9	5	9	0.78	0.56	0.13
CL 22	7	9	8	9	0.78	0.89	0.87
CL 31	9	9	2	9	1.00	0.22	3.05
CL 32	8	9	7	9	0.89	0.78	3.05
CL 34	7	9	3	9	0.78	0.33	3.05
CL 37	9	9	11	9	1.00	1.22	3.05

References:

1. Bshary, R. & Grutter, A. S. Punishment and partner switching cause cooperative behaviour in a cleaning mutualism. *Biol. Lett.* **1**, 396–399 (2005).

2. Bshary, R. & Grutter, A. S. Image scoring and cooperation in a cleaner fish mutualism. *Nature* **441**, 975–978 (2006).
