# Supplementary material for: Brain morphology predicts social intelligence in wild cleaner fish
Source: Nat Commun. 2020 Dec 21;11:6423. doi: 10.1038/s41467-020-20130-2 (PMC7752907; doi:10.1038/s41467-020-20130-2)
Supplement: Supplementary file 3 — Reporting Summary [file 41467_2020_20130_MOESM3_ESM.pdf]

nature research

Corresponding author(s): Zegni Triki

Last updated by author(s): Nov 3, 2020

Reporting Summary

Nature Research wishes to improve the reproducibility of the work that we publish. This form provides structure for consistency and transparency in reporting. For further information on Nature Research policies, see our Editorial Policies and the Editorial Policy Checklist.

Statistics

For all statistical analyses, confirm that the following items are present in the figure legend, table legend, main text, or Methods section.

n/a	Confirmed	
☐	☒	The exact sample size (*n*) for each experimental group/condition, given as a discrete number and unit of measurement
☐	☒	A statement on whether measurements were taken from distinct samples or whether the same sample was measured repeatedly
☐	☒	The statistical test(s) used AND whether they are one- or two-sided<br>*Only common tests should be described solely by name; describe more complex techniques in the Methods section.*
☐	☒	A description of all covariates tested
☐	☒	A description of any assumptions or corrections, such as tests of normality and adjustment for multiple comparisons
☐	☒	A full description of the statistical parameters including central tendency (e.g. means) or other basic estimates (e.g. regression coefficient) AND variation (e.g. standard deviation) or associated estimates of uncertainty (e.g. confidence intervals)
☐	☒	For null hypothesis testing, the test statistic (e.g. *F*, *t*, *r*) with confidence intervals, effect sizes, degrees of freedom and *P* value noted<br>*Give P values as exact values whenever suitable.*
☒	☐	For Bayesian analysis, information on the choice of priors and Markov chain Monte Carlo settings
☐	☒	For hierarchical and complex designs, identification of the appropriate level for tests and full reporting of outcomes
☐	☒	Estimates of effect sizes (e.g. Cohen's *d*, Pearson's *r*), indicating how they were calculated

Our web collection on statistics for biologists contains articles on many of the points above.

Software and code

Policy information about availability of computer code

Data collection	*Provide a description of all commercial, open source and custom code used to collect the data in this study, specifying the version used OR state that no software was used.*
Data analysis	Analyses reported in this article can be reproduced using code provided by Triki et al. (https://doi.org/10.6084/m9.figshare.7415576).m9.figshare.7415576).

For manuscripts utilizing custom algorithms or software that are central to the research but not yet described in published literature, software must be made available to editors and reviewers. We strongly encourage code deposition in a community repository (e.g. GitHub). See the Nature Research guidelines for submitting code & software for further information.

Data

Policy information about availability of data

All manuscripts must include a data availability statement. This statement should provide the following information, where applicable:

- Accession codes, unique identifiers, or web links for publicly available datasets
- A list of figures that have associated raw data
- A description of any restrictions on data availability

Source data are provided with this paper and they are archived at Figshare data repository by Triki et al. (https://doi.org/10.6084/m9.figshare.7415576).

Field-specific reporting

Please select the one below that is the best fit for your research. If you are not sure, read the appropriate sections before making your selection.

☒ Life sciences ☐ Behavioural & social sciences ☐ Ecological, evolutionary & environmental sciences

For a reference copy of the document with all sections, see nature.com/documents/nr-reporting-summary-flat.pdf

Life sciences study design

All studies must disclose on these points even when the disclosure is negative.

Sample size	For the fish survey part, we collected fish censuses on transect lines of 30 m and 5 m width: in three sites we collected 10 transects each, and in one site we collected 7 transects. Number of transects was chosen based on previous studies (see Triki, Z. et al. Biological market effects predict cleaner fish strategic sophistication. Behavioral Ecology 30, 1548–1557 (2019) showing that such sample size can still yield applicable results.<br><br>We first collected 40 cleaner fish from four different reef sites around Lizard Island, Great Barrier reef. All 40 fish were tested for their cognitive performance. Upon accomplishment of the task, and based on their performance, we selected ten high-performers and the ten low-performers for the brain analyses. It is noteworthy to justify the use of 20 cleaners instead of killing all the 40 tested females. Our field site at Lizard Island suffered from a severe decline in fish densities following consecutive environmental perturbations, like cyclones and coral bleaching. The directors of the Lizard Island Research Station, Dr Anne Hogget and Dr Lyle Vail are concentrating efforts to sustain coral reef and fish communities recovery. After consulting with them regarding this project, it was agreed to sample 20 fish instead of 40, and thus returning the other 20 to their home reef to reduce disturbances due to absence of cleaners on coral reefs
Data exclusions	there were no data exclusion from the fish survey data.<br><br>Two brain samples were excluded due to erroneous weight reading: Three brain parts samples that belong to two different fish had an error while reading tissue weight, and as it was not possible to weigh them again as they were already being transferred to the PFA solution. The two brain samples were excluded from brain part sizes analyses, but were included in the cell count analyses since we were still able to count the cells of the parts without mass information.
Replication	The collected data from the fish survey were not replicated. The data were collected in a single event. We were not able to replicate the process given time constraint during the field trip. We needed to start collecting fish from these sites to start the laboratory tests. For this reason, any replicate after fish removal will not be reliable as we deliberately may have changed cleaner fish densities by removing 40 cleaners from the study sites.<br><br>In the cognitive task, fish were tested in up to 200 trials. we already set a strict success criterion. That is, every fish needed to significantly pass the test to be categorized as successful learner. To solve the task, a cleaner fish had to show a significant preference towards the visitor plate, which consisted of a score of either: nine or more successful choices out of a session of 10 trials; two consecutive eight successful choices out of sessions of 10 trials each; three consecutive seven successful choices out of sessions of 10 trials each.
Randomization	There were no need to allocate fish into experimental groups. All fish received the same task.<br><br>Randomization was needed during the cognitive task: the decoration, the spatial location (i.e., left or right) and the status of the plexiglas plates (i.e., surrogates for visitor or resident clients) were counterbalanced.
Blinding	Blinding the identity of fish (i.e., site of capture) was not possible as the experimenters were the ones who caught the fish from these reef sites.<br><br>Brain measurement were done blindly regarding the identity (i.e., performance and site) of the samples.

Reporting for specific materials, systems and methods

We require information from authors about some types of materials, experimental systems and methods used in many studies. Here, indicate whether each material, system or method listed is relevant to your study. If you are not sure if a list item applies to your research, read the appropriate section before selecting a response.

Materials & experimental systems

n/a	Involved in the study
☐	☒ Antibodies
☒	☐ Eukaryotic cell lines
☒	☐ Palaeontology and archaeology
☐	☒ Animals and other organisms
☒	☐ Human research participants
☒	☐ Clinical data
☒	☐ Dual use research of concern

Methods

n/a	Involved in the study
☒	☐ ChIP-seq
☒	☐ Flow cytometry
☒	☐ MRI-based neuroimaging

Antibodies

Antibodies used

Anti-NeuN (rabbit) Antibody, Cy3 Conjugate ABN polyclonal, Merck. Catalogue number: ABN78C3

Validation

"ABN78 is a rabbit polyclonal version of the Anti-NeuN, clone A60 (MAB377), NeuN is a highly characterized and cited mouse monoclonal antibody that specifically recognizes the DNA-binding, neuron-specific protein NeuN, which is present in most CNS and PNS neuronal cell types of all vertebrates tested. NeuN protein distributions are apparently restricted to neuronal nuclei, perikarya and some proximal neuronal processes in both fetal and adult brain although, some neurons fail to be recognized by NeuN at all ages: INL retinal cells, Cajal-Retzius cells, Purkinje cells, inferior olivary and dentate nucleus neurons, and sympathetic ganglion cells are examples. Immunohistochemically detectable NeuN protein first appears at developmental timepoints that correspond with the withdrawal of the neuron from the cell cycle and/or with the initiation of terminal differentiation of the neuron. Immunoreactivity appears around E9.5 in the mouse neural tube and is extensive throughout the developing nervous system by E12.5. Strong nuclear staining suggests a nuclear regulatory protein function; however, no evidence currently exists as to whether the NeuN protein antigen has a function in the distal cytoplasm or whether it is merely synthesized there before being transported back into the nucleus. No difference between protein isolated from purified nuclei and whole brain extract on immunoblots has been found." from the manufacturer website: https://www.merckmillipore.com/SE/en/product/Anti-NeuN-rabbit-Antibody-Cy3-Conjugate,MM_NF-ABN78C3?ReferrerURL=https%3A%2F%2Fwww.google.com%2F&bd=1#anchor_Description

Animals and other organisms

Policy information about studies involving animals; ARRIVE guidelines recommended for reporting animal research

Laboratory animals

No laboratory animals were used in this study

Wild animals

We studied the cleaner fish Labroides dimidiatus. We captured adult female cleaners (total length TL: mean ± SD, 7.43 ± 0.61 cm) at Lizard Island. Scuba divers captured cleaners with barrier nets (2 m x 1 m, 5 mm mesh) and hand nets. Fish were then transported to Lizard Island facilities in buckets filled with seawater (20 L buckets). At the end of the laboratory tasks, 20 female cleaners were sacrificed by a rapid cervical transection. The remaining 20 female cleaners were returned and released in their site of capture.

Field-collected samples

Scuba divers captured cleaners with barrier nets (2 m x 1 m, 5 mm mesh) and hand nets. At Lizard Island Research Station facilities, all fish were individually housed in glass aquaria (62 cm x 27 cm x 37 cm) and provided with PVC pipes (10 cm x 1 cm) as shelters. All fish were allowed an acclimation period of at least 14 days before proceeding with the laboratory experiments. Fish were fed daily with a paste of mashed prawn smeared on Plexiglas plates (8 x 15 cm). During Laboratory experiments, cleaners received food from the trials from 8:00 to 17:00. The housing aquaria were exposed to a photo-period of 11 hours light and 14 hours dark. The aquaria had a running seawater pumped directly from the ocean. According to the DataCite
(Australian Institute of Marine Science (AIMS) ; Integrated Marine Observing System (IMOS) (2020): Lizard Island Weather Station installed on Sensor Relay Pole 2. Australian Institute of Marine Science (AIMS).dataset.
http://apps.aims.gov.au/metadata/view/6adcb039-d581-4296-bbb0-895f25977ef9), the water temperature during the data collection (July-August 2018) was on average 24°C.
All fish were in good health throughout the period they were held in captivity. There were no need for an end-of-experiment protocol which states as termination if fish shows sever illness symptoms.

Ethics oversight

The Animal Ethics Committee of the Queensland government (DAFF) approved the project under the number CA 2017-05-1063.

Note that full information on the approval of the study protocol must also be provided in the manuscript.
