# Supplementary material for: Brain morphology predicts social intelligence in wild cleaner fish
Source: Nat Commun. 2020 Dec 21;11:6423. doi: 10.1038/s41467-020-20130-2 (PMC7752907; doi:10.1038/s41467-020-20130-2)
Supplement: Supplementary file 2 — Peer Review File [file 41467_2020_20130_MOESM2_ESM.pdf]

Reviewer #1 (Remarks to the Author):

The present study “Trade-off between forebrain and cerebellum size is associated with cleaner fish cognitive performance as a function of population density” by Triki and colleagues takes advantage of the unique biology of cleaner fish to explore associations between brain characteristics, ecological context, and performance of a complex task. I find the innovative utilization of such a fascinating model system compelling. The results interesting and complex. At present my main concerns with the manuscript are in the interpretation of the findings and the need for clarification of a few major and minor points.

First, the authors couch their findings in the context of adaptive evolution in brain size (see especially introduction and last paragraph of discussion). However, there are also mentions of developmental influences, behavioral plasticity, and decision-making throughout the text. At present I find the authors’ interpretation of the relationship between these phenomena muddled and this should be clarified. Is the assumption that increased brain size makes fish able to perform better on a cognitive task or that increased brain size is associated with better ‘behavioral switching’ to adjust behavior for current social environment? Do they authors assume that these traits are fixed? Developmentally determined? Acquired through learning throughout life? It seems to me that these alternative scenarios make quite different predictions about the association between brain metrics, population density and performance. For example, under the assumption of adaptive evolution in brain size associated with social competence, shouldn’t we expect fish from high density populations to show on average better cognitive performance?

Second, and related to the above comment, additional details should be included regarding the biology of the fish to contextualize the relationship between individual experience and population density. Do fish experience stable populations densities over their lifetime? Or are populations sizes variable and/or can fish experience different population densities by moving between sites? How long to these animals live? Is the expectation that fish that experience high density communities are more socially competent? Or would fluctuating densities be more likely to select for fish that flexibly adjust behavior to exhibit an optimal strategy?

Both of the above would be clarified through additional details regarding the sampled fish. Are the ten high-performing fish from higher densities? Or is average density equal between high- and low-performers? Specifying these details and/or including basic summary metrics in a table seems critical.

Third, there are repeated statements about high-performers having relatively smaller or larger forebrains than low-performers (lines 131-133, 151-152, data from Fig. 2a). These statements are inappropriate without a posthoc test to explicitly confirm this pattern (e.g. it is possible to have a significant interaction effect but no significant simple effects between groups). In addition, if these conclusions are made for the forebrain parallel interpretations should be included for the cerebellum (data from Fig. 2c).

Fourth, the data do suggest that fish with smaller cerebella also have high cell density in the cerebellum. This suggests there could also be a change in size without a change in functionality (e.g. if cell number is maintained). This alternative should also explicitly included in the discussion (paragraph starting Line 236).

Fifth, in the discussion the authors suggest that brain size differences are driven by changes specifically in neuropil. Is there evidence that this is broadly the case? If so, please provide references. If not, discussion of alternative interpretations is necessary.

Finally, given the complex somewhat subtle nature of the results I encourage the authors to include estimates of effect sizes with their results.

MINOR COMMENTS

Line 17: Contains typo. Remove second ‘that’.

Lines 47-49: Seems the work on guppy brain size evolution by Kortschal and collegues would be relevant here.

Line 80: For clarity, I recommend replacing “it suggests” with “these observations suggest”.

Lines 95 – 97: Please clarify here the why cleaner fish can be less choosy at low densities (currently not explained until discussion).

Lines 119-121: Was scaling done to either or both metrics? Methods seem to suggest both.

Line 127: Proportion of brain part to whole brain? Please clarify.

Lines 129-129: Including metrics regarding group-level variation would also be informative here.

Lines 142-143: Please note that data for forebrain and cerebellum are presented in the Supplementary Materials.

Line 145: Please clarify what is meant by “cell count proportions”. Counts as a proportion of what?

Line 151: Many of the details included in this paragraph seem more appropriate for the discussion.

Line 179: Not clear what ‘for those’ refers to. The brain areas or the individual fish?

Lines 310-312: It is stated that the location and status of the plates were counterbalanced. Is this between individuals, or were some aspects (e.g. plate position) also varied between trials?

Line 371 & 374: The size or the weight of the brain part? I find ‘size’ misleading given that all conclusions are based on weight.

Table 1: N values suggest some sample were removed. Please explain in methods.

Table 1: It would be helpful to include an explanation of how metrics in the table were calculate in the body of the methods rather than only as part of the Table legend.

Figures 2-4: Please add R2 values to the graphs (as are provided in supplemental). Also please indicate which effect the p-values refer to (i.e. interaction effect, main effect, etc).

Figure 4: For consistency, I think it would be valuable to include a graph of the forebrain cell density data here as well

Figure S1: Please explain the colors.

Table S1: The cell counts are per ml. Is this or brain tissue? Cell suspension?

Reviewer #2 (Remarks to the Author):

On the whole this is a nice experiment examining the effect of social environment on brain morphology in cleaner fish. There are some odd statements about a lack of similar studies but there are several that spring to mind (cichlids -Nick Kolm, Winberg and others- and our own work in gobies). Very little of that work is mentioned which is odd. The former specifically focus on the social environment although they did not follow up with cognition tests. The latter focus on physical structure on brain morphology and cognition. It would be fair to say the few have looked at social environment, brain morphology and cognition.

The sample size is pretty small for the brain analysis, but there seems to be good ethical reasons for that. There were also some unfortunate accidents which means that cell counts were conducted but not neuron number. It would have been great to estimate synaptic number/density as well. Nonetheless, the relative lobe size analysis yielded some interesting findings.

One possible confound is that the fish brain may have responded to the training regime. Fish brains are crazy plastic! The only way to avoid that is to examine brains from representative populations taken directly from the wild. But as we see here, even population identity was not a predictor of brain size nor cognitive skills. Rather high performing individuals were identified from both populations and their brain morphology varied with social density.

While I like the social competence hypothesis, I rather think that demand for cognition likely drives investment in brain tissue and thus improves cognition in given domains during ontogeny. This is a more unifying explanation for these data and others.

Specific comments
L49 and L60: Not sure I agree with this statement. My lab has a series of paper examining cognition and brain morphology in intertidal gobies and how it varies with environmental complexity (See a series of papers by White & Brown, Carbia & Brown etc). Most of the animals are drawn from the wild, but we have also manipulated rearing environment in a captive setting.
L109: It’s not clear that you looked at all lobes of the brain. You should probably explicitly state that other parts of the brain serve as a “control” for your social brain hypothesis.
L127: In general, I think this is a far better approach since it can tell you what parts of the brain the animal is preferentially investing in. See our goby papers for reference.
L131: in other words there was an interaction between pop density and performance.
L134: we found similar trade-offs in gobies (https://doi.org/10.1159/000380875)
L207: we have shown that environmental enrichment in the captive environment also increases cognitive performance (https://doi.org/10.1007/s10071-018-1225-8)
L209: this is a bit of a chicken and the egg argument. Given what we know about how incredibly plastic the fish brain is, it seems highly likely to me that fish will only develop enough brain capacity to solve local problems. Which is a slightly different argument than the one you pose here. In other words, there must be a need for adaptive plasticity to generate the brain capacity to cope with that need.
L221: Indeed, it’s a shame you didn’t look at connectivity (synapses). Sounds like a future project.
L231: Yes, in fact you are making a pretty dodgy assumption that the only thing that varies between populations is social structure, but in fact there are likely to be other important environmental differences, such as structural complexity. You make mention of this in your introduction but don’t acknowledge it here.
L244: I guess the question arises here as to whether the reduction in cerebellum is adaptive or is it the “sacrificial lamb”? The animals need to devote more energy (and space) to the forebrain so something has to give.
L253: Some of the papers examining brain miniaturization in ants may be of interest here.
L274: Do you mean 2.5m either side of the transect?
L309: This section is a little unclear. Did all fish really undertake 200 trials or did they switch to reversal after reaching criteria?

L326: It sounds like you did not use the reversal data. Is that the case?
L331: presumably none of the poor performers made it to the reversal phase. Or did they? There is not much point in running reversals if they didn't learn the first task. I wouldn't bother mentioning this, it just takes up space. It would be good to know the total number of trails the fish were exposed to. It may well be that there was some plasticity due to training (I think Alex Kortschal has investigated this)
L364: how unfortunate
L377: This is my preferred method as mentioned previously.

Culum

Reviewer #3 (Remarks to the Author):

This manuscript reports the results of a study aiming to identify interactions between social environment, brain morphology and cognitive performance in natural populations in cleaner fish. Sampling from four different reef sites, and utilizing an ephemeral reward task, individual fish were tested on task performance with both brain morphology and social density being used to predict learning performance. The performance measure was binary, with fish either learning the task within 200 trials or not. The learning criterion was based on performance over 10 trial sessions. While no relationship was found when looking at measures of the midbrain or brain stem, significant interactions were found involving both the forebrain and cerebellum with social density. Thus a trade-off was found such that those fish that completed the task tended to have larger forebrains and smaller cerebellums if they were in high density environments but the opposite relationship when in low density environments. Among the fish that did not complete the task, no relationship was found between social density and brain morphology. Given these somewhat counterintuitive effects, the authors then examined the ecological relevance of different decisions rules within the ephemeral task as it pertains to social density differences. From this it was concluded that the results suggest that socially competent fish have larger brains, performing the task using the decision rule most adaptive for their social environment (i.e., reach criterion when from high density environments; do not reach criterion within 200 trials if from low density environment).

I find the topic to be of particular scientific merit as there is a definite need for more studies examining the mechanisms driving individual variation in cognition/task performance, particularly in wild populations, as well as studies trying to explore the ecological factors involved. The authors do a nice job of highlighting how this current study expands upon the literature, and stating their predictions. The paper is generally well written though there are some areas where grammar issues/minor typos make it a little difficult to read. Additionally, while I appreciate the authors wanting to be provide as much information about the study as possible and explain rationale (e.g., reduction of Type 1 error) I do think the paper could be streamlined quite a bit to improve overall clarity and readability. See comments below for some examples.

Overall, I found the study to be well designed, and given conservation concerns the sample size is understandable. My main concerns have to do with some of the set up in the introduction and with the authors' interpretation of their results which I feel need to be addressed. First, in the introduction the study is set up with discussion of evolutionary comparative analysis and plasticity, but none of this is really addressed by the study itself. We are told nothing about the underlying genetic differentiation of these populations, and as presented it is unclear if the measured variation is actually due to any plastic response. Discussion of ontogeny is then continued in the discussion, but again the role of development on brain morphology and task performance is not examined in this study. Even the studies that are referenced do not seem to directly address this concern. I would suggest reframing the study away from these topics unless you feel you can better connect them with the current study.

The second issue has to do with the interpretation of the interaction between brain morphology (forebrain and cerebellum) and social environment. While there is a significant interaction effect indicating that the slopes of learners and non-learners differ, alone this doesn't say anything about how these groups differ at different densities? Were any post-hoc contrasts done? Without showing these contrasts, the authors need to watch out for making comparisons between groups (e.g., Lines 151-152), and be a little careful about how they relate these findings to their initial predictions and in their justification for setting up the social competence narrative.

The final issue has to do with the social competence framework itself. While the rationale is clearly articulated for why you would expect to see social competence and task performance be in line with one another at higher densities, why you would expect worst performance from socially competent fish from low social densities conditions is still unclear to me. How is getting less food (consequence of incorrect trials) optimal in this case. Also, if visitors have no choice in low densities and so have to stick around, is this task really relevant to these conditions? Visitor cues should simply be irrelevant, but this wouldn't mean it would be optimal to not follow cues during the ephemeral reward task. Particularly if you want to argue that larger brained fish that are presumably more "intelligent" or socially competent are better able to adapt plastically to local conditions (lines 210-211) then I think you would expect them to adapt to the learning conditions of the task faster. While it is interesting that you see a significant difference in brain morphology when you looked to examine social competence, more work needs to be done to build the argument that manipulating the data in this way is warranted biologically. While I would like to see all of these concerns addressed before I would consider this study ready for publication, given the overall conclusions from the paper I believe addressing this final concern is the most critical.

Detailed comments:

Lines 80-81: Unclear if this is saying these differences have evolved amongst populations or are due to developmental plasticity

Line 94: A little hard to tell from the figures but it seems like success task success was generally similar across social density groups? Was this true? Maybe summarize number of successful fish across density groups in the Results?

Line 101: Why only females? Sex effects?

Line 106: This wording is confusing. On my initial read it seemed to imply 20 individuals from each of 4 sites (so 80 total)

Paragraph starting with line 151: Initially this interpretation of results seem better suited for the discussion. I understand that this is needed to set up the follow up analysis, but is there maybe a better way to set this up in the intro so that it doesn't seem to come out of nowhere in the middle of the results? Feels out of place here.

Lines 151-152: As presented the stats don't say anything about the comparisons across performers and nonperformers being significant. I understand the authors simply point to "relative" differences of the observed fish but need to be careful about overinterpreting results about group comparisons.

Line 170: Why would it be "optimal" to ignore visitors and get less food in low density context? Are you suggesting costs to identifying visitors?

Lines 178-182: I found this sentence awkwardly written. I would suggest rewriting it.

Lines 209-211: But then wouldn't the bigger brained fish show an ability to adapt to the test environment? Instead the smaller brained fish are getting more food.

Line 217: With the slightly different section order of this publication I would reference your methods here so the reader knows that the reason for this missing data will be explained there.

Line 232: “captures” instead of “capture”

Line 304: 200 trials per fish for 10 days could be interpreted as 2000 trials total for each fish. Maybe reword.

Lines 311-312: In what way were things counterbalanced? Between trials, between fish? I assume between fish such that the pattern and status of the plates differed between fish but were consistent for that fish over all of their trials, but was side also consistent for each fish? In other words was the “visitor plate” always on the right for Fish A?

Line 344: Maybe reorder your figures for that Fig 1 isn’t being referenced after other figures.

Lines 363-366: This should be referenced above in the discussion, or maybe moved to the discussion when you talk about not having neuron numbers.

Lines 386-387: Example of a line that could be cut since it is redundant with text found in the results section.

Line 414-415: What were these behavioral observations for? The audience effect mentioned above? Feel this can should be removed along with discussion of audience effects if not used for the analysis of this study. Similarly I think mentions of the reversal learning phase could also be removed.

Reviewer #1:

General Comments

The present study “Trade-off between forebrain and cerebellum size is associated with cleaner fish cognitive performance as a function of population density” by Triki and colleagues takes advantage of the unique biology of cleaner fish to explore associations between brain characteristics, ecological context, and performance of a complex task. I find the innovative utilization of such a fascinating model system compelling. The results interesting and complex. At present my main concerns with the manuscript are in the interpretation of the findings and the need for clarification of a few major and minor points.

First, the authors couch their findings in the context of adaptive evolution in brain size (see especially introduction and last paragraph of discussion). However, there are also mentions of developmental influences, behavioral plasticity, and decision-making throughout the text. At present I find the authors’ interpretation of the relationship between these phenomena muddled and this should be clarified.

Reply 1:

We have edited the introduction following the reviewers’ comments. Overall, we believe that it reads better than in the previous version. We also made sure that the transition between the different paragraphs and statements became smoother.
This also applies to the last sentence in the last paragraph of the discussion.

Is the assumption that increased brain size makes fish able to perform better on a cognitive task or that increased brain size is associated with better 'behavioral switching' to adjust behavior for current social environment? Do they authors assume that these traits are fixed? Developmentally determined? Acquired through learning throughout life? It seems to me that these alternative scenarios make quite different predictions about the association between brain metrics, population density and performance. For example, under the assumption of adaptive evolution in brain size associated with social competence, shouldn't we expect fish from high density populations to show on average better cognitive performance?

Reply 2:

In the revised manuscript, we have added substantial information on the matter in the introduction. The scenario that we suggest is that of "learning throughout life". This scenario is the best supported by previous studies and knowledge about the ecology of the cleaner fish.

Second, and related to the above comment, additional details should be included regarding the biology of the fish to contextualize the relationship between individual experience and population density. Do fish experience stable populations densities over their lifetime? Or are populations sizes variable and/or can fish experience different population densities by moving between sites?

Reply 3:

Following the reply above (Reply 2). The text added to the introduction addresses these matters in detail.

How long to these animals live?

Reply 4:

We have now added information on the lifespan of cleaner fish which is a maximum of about 5 years according to the study by Eckert [1]. Line 128.

Is the expectation that fish that experience high density communities are more socially competent? Or would fluctuating densities be more likely to select for fish that flexibly adjust behavior to exhibit an optimal strategy?

Reply 5:

Given that our experimental design chose explicitly high and low performers from different densities (Line 142-143), we wouldn't expect to have a main effect of cleaner density on performance. However, we used density in the statistical models (Line 151-152) to test whether changes in brain morphology (brain part sizes, and cell counts) have a main effect on performance or whether it predicts performance as a function of the density of cleaners ("brain X population density" interaction effect).

We have now added this clarification to the discussion. The sentence reads as

*follows: "**Given that our experimental design selected explicitly high and low performers from different densities, there could not be a main effect of cleaner density on performance.**" Line 248-249*

Furthermore, our data show that cleaners with the larger forebrains are more socially competent (Fig. 4a). The optimal behaviour in question is different between sites depending on the ecological conditions (i.e., cleaner density). So, we cannot answer the question of whether social competence increases as cleaner density increases. More generally, and in addition to the findings by Triki et al.[2], we do not know what cleaners at higher densities do with larger forebrains.

Both of the above would be clarified through additional details regarding the sampled fish. Are the ten high-performing fish from higher densities? Or is average density equal between high- and low-performers? Specifying these details and/or including basic summary metrics in a table seems critical.

Reply 6:

We agree that the information was missing in the main text. We have now added detailed information about the sampled fish in a Supplementary table (S1). The average cleaner density for high- and low-performers was 3.96 and 3.32 (cleaner per 100 m2), respectively.

Third, there are repeated statements about high-performers having relatively smaller or larger forebrains than low-performers (lines 131-133, 151-152, data

from Fig. 2a). These statements are inappropriate without a posthoc test to explicitly confirm this pattern (e.g. it is possible to have a significant interaction effect but no significant simple effects between groups).

Reply 7:

Given that the interaction is between two continuous variables, there is no straightforward way to compare groups. Besides, our sample size is relatively low at every reef site (or density) which makes group comparisons not feasible. Nevertheless, we have used an R package, that depicts the interaction effect in sort of a post hoc plot (Supplementary Fig. S2) (Line 454-457). This figure shows that high-performing individuals had relatively larger forebrains and smaller cerebella when living in high population densities, but smaller forebrains and larger cerebella when living in low densities.

*Nevertheless, we agree that our previous statement implied group differences that we did not test directly. We have now fixed these statements throughout the text accordingly. Most of these statements read as follows: "**forebrain size of high-performing individuals tested in an ephemeral reward task covaried positively with cleaner density, while cerebellum size covaried negatively with cleaner density**." Line 27-29.*

In addition, if these conclusions are made for the forebrain parallel interpretations should be included for the cerebellum (data from Fig. 2c).

Reply 8:

We have included the cerebellum whenever we mentioned the forebrain relationship with cleaner density in predicting performance.

Fourth, the data do suggest that fish with smaller cerebella also have high cell density in the cerebellum. This suggests there could also be a change in size without a change in functionality (e.g. if cell number is maintained). This alternative should also explicitly included in the discussion (paragraph starting Line 236).

Reply 9:

This actually has been already said explicitly in the discussion, see Line 279-282 in the new version.

Fifth, in the discussion the authors suggest that brain size differences are driven by changes specifically in neuropil. Is there evidence that this is broadly the case? If so, please provide references. If not, discussion of alternative interpretations is necessary.

Reply 10:

We have added some relevant references to support our argument. Ref 56 and

Ref 57 in the main text.

Finally, given the complex somewhat subtle nature of the results I encourage the authors to include estimates of effect sizes with their results.

Reply 11:

We have now added effect size (Cohen's d) to the results Table 1.

Minor comments:

Line 17: Contains typo. Remove second 'that'.

Reply 12:

We corrected the typo.

Lines 47-49: Seems the work on guppy brain size evolution by Kortschal and collegues would be relevant here.

Reply 13:

We have now added the following sentence: ***"Indeed, artificial selection on brain size can result in large-brained populations with higher cognitive abilities, such as learning in mice (Wimer and Prater 1966) and guppies (Kotrschal et al. 2013; Buechel et al. 2018)"*** *with reference to studies showing a link between cognitive abilities and artificial selection on brain size. Line 47-49.*

Line 80: For clarity, I recommend replacing "it suggests" with "these observations suggest".

Reply 14:

This sentence in question has been deleted in the revised version of the manuscript. Although, in another similar sentence we have applied this suggestion and replaced "it suggests" with "these observations suggest". Line 134.

Lines 95 – 97: Please clarify here the why cleaner fish can be less choosy at low densities (currently not explained until discussion).

Reply 15:

Based on the reviewers' comments, we have now edited the introduction and added some text for clarity. We have now added a substantial clarification based

on data why cleaner fish can be less choosy and prioritize the visitor clients less at low densities.

Lines 119-121: Was scaling done to either or both metrics? Methods seem to suggest both.

Reply 16:

We have removed the technical details of the two scaling methods from the Results, but kept it in the Methods section (Line 397-402). We prefer the proportion calculation as a scaling method, which is also an encouraged method (see Column Brown's comments below). Nevertheless, the two scaling methods used in this study did not provide different outcomes, such that changes in proportions was also accompanied by changes in brain measurements scaled to body size (Supplementary Figure S1).

Line 127: Proportion of brain part to whole brain? Please clarify.

Reply 17:

We deleted that sentence.

Lines 129-129: Including metrics regarding group-level variation would also be

informative here.

Reply 18:

Both our explanatory variables are continuous predictors. Therefore, we cannot provide group level details (Line 454-457). Nevertheless, and following previous comments, we have added a Supplementary Table S1 to provide further information on the collected fish.

Lines 142-143: Please note that data for forebrain and cerebellum are presented in the Supplementary Materials.

Reply 19:

The information on the forebrain and cerebellum is not repetitive in the supplementary material, but rather providing further information on the relationship between body size and brain region measurements.

Line 145: Please clarify what is meant by “cell count proportions”. Counts as a proportion of what?

Reply 20:

We have corrected this throughout the text and used "cell proportion" instead. We meant with cell proportions as the number of cells from a brain region divided by the total brain cells (Line 180).

Line 151: Many of the details included in this paragraph seem more appropriate for the discussion.

Reply 21:

The referred details have been moved up to the introduction for more clarity.

Line 179: Not clear what 'for those' refers to. The brain areas or the individual fish?

Reply 22:

We have now clarified this sentence. We meant with "those" the significant results from the initial analyses of cleaner performance (Line 197-198).

Lines 310-312: It is stated that the location and status of the plates were counterbalanced. Is this between individuals, or were some aspects (e.g. plate position) also varied between trials?

Reply 23:

We have made it clear in the text that the status and plate colour were different between individuals, but the plate position was different between trials (Line 344-349).

Line 371 & 374: The size or the weight of the brain part? I find 'size' misleading given that all conclusions are based on weight.

Reply 24:

We have replaced "size" with "weight" in Line 144, and Line 397 when we describe the methods of size estimation.

Table 1: N values suggest some sample were removed. Please explain in methods.

Reply 25:

*We do have an explanation for the missing values in the Methods section. The sentence reads as follows: "**Three brain part samples that belong to two different fish had an error while reading tissue weight, and as it was not***

***possible to weigh them again as they were already being transferred into the PFA solution. Nevertheless, we were able to estimate cell counts from these three samples**." Line 374-377.*

Table 1: It would be helpful to include an explanation of how metrics in the table were calculate in the body of the methods rather than only as part of the Table legend.

Reply 26:

We have added the required information to the Methods section. Line 447-451.

Figures 2-4: Please add R2 values to the graphs (as are provided in supplemental). Also please indicate which effect the p-values refer to (i.e. interaction effect, main effect, etc).

Reply 27:

We have added the R2 values to the main graphs.

Figure 4: For consistency, I think it would be valuable to include a graph of the forebrain cell density data here as well

Reply 28:

*We believe that in the revision it becomes clearer why we restricted the social competence analyses to these three measurements. The argument as stated in the text reads as follows: "**With the new labelling, we reran the analyses only for the brain region measurements showing a significant interaction with population density (i.e., forebrain size, cerebellum size, and cerebellum cell density) in predicting performance in the previous analyses. With the new analyses, we tested predictions for social competence instead**." Line 196-200.*

Figure S1: Please explain the colors.

Reply 29:

We have now added a color bar to the figure S1 showing the associated Pearson's correlation coefficient.

Table S1: The cell counts are per ml. Is this or brain tissue? Cell suspension?

Reply 30:

We have now added information about the cell counts per ml to the Table S1

*legend: "**For cell counts, every brain part was reduced to a suspension of cells in 1 mL of PBS solution (see Methods).**"*

Reviewer #2: *Dr. Culum Brown*

General comments:

On the whole this is a nice experiment examining the effect of social environment on brain morphology in cleaner fish. There are some odd statements about a lack of similar studies but there are several that spring to mind (cichlids -Nick Kolm, Winberg and others- and our own work in gobies). Very little of that work is mentioned which is odd. The former specifically focus on the social environment although they did not follow up with cognition tests. The latter focus on physical structure on brain morphology and cognition. It would be fair to say the few have looked at social environment, brain morphology and cognition.

Reply 31:

We have added the relevant citations to the revised manuscript. Our statement in fact states exactly what the reviewer is suggesting. Despite that some research looked at the relationship between either brain morphology, ecology, or cognition, this wasn't carried out on the very same individuals, but instead on the species or population level.

*For more clarity and to make our statement as precise as possible, we rewrote this sentence to read as follows: "**Yet, assessing simultaneously the ecological***

***factors, brain morphology, and cognitive performance at the intra-specific level has been rarely done**." Line 56-58.*

The sample size is pretty small for the brain analysis, but there seems to be good ethical reasons for that. There were also some unfortunate accidents which means that cell counts were conducted but not neuron number. It would have been great to estimate synaptic number/density as well. Nonetheless, the relative lobe size analysis yielded some interesting findings.

Reply 32:

We agree that there were some unfortunate events with the data collection. We have now added the effect size (Cohen's d) (see Table 1) to provide further information about the data and the analyses.

One possible confound is that the fish brain may have responded to the training regime. Fish brains are crazy plastic! The only way to avoid that is to examine brains from representative populations taken directly from the wild. But as we see here, even population identity was not a predictor of brain size nor cognitive skills. Rather high performing individuals were identified from both populations and their brain morphology varied with social density.

Reply 33:

Given the findings by Fong et al. [3]*, it is unlikely that the training regime impacted brain plasticity. We have now added this statement to the introduction.* *Line 153-154.*

While I like the social competence hypothesis, I rather think that demand for cognition likely drives investment in brain tissue and thus improves cognition in given domains during ontogeny. This is a more unifying explanation for these data and others.

Reply 34:

As explained in the first paragraph of the Discussion, one needs to take into account the ecology of individual in order to interpret its performance. This has been intensively reviewed by Thornton and Lukas [4]*.*

We do not know what are the exact cognitive mechanisms that underlie social competence. But social competence is cognitively demanding: The need to acquire, encode, store and recall information about the social environment must have underlying neural mechanisms (see review by Varela et al. [5]*). Here, we find such a link at the intraspecific level in cleaner fish.*

Specific comments

L49 and L60: Not sure I agree with this statement. My lab has a series of paper examining cognition and brain morphology in intertidal gobies and how it varies with environmental complexity (See a series of papers by White & Brown, Carbia & Brown etc). Most of the animals are drawn from the wild, but we have also manipulated rearing environment in a captive setting.

Reply 35:

We agree that this statement was not clear enough (Line 56-57). We have now tried to clarify it in the new revision. However, the suggested studies by the reviewer, although they deal with the same population, the data was not dependent. That is, linking cognition, brain morphology and environmental complexity was not performed on the very same individuals. Nevertheless, from the literature, one can say that these are the closest to examine such links at the population level in fish. We have now included some of the relevant citations in the revision.

L109: It's not clear that you looked at all lobes of the brain. You should probably explicitly state that other parts of the brain serve as a "control" for your social brain hypothesis.

Reply 36:

*We have added the following: "**We assessed in the selected cleaners the weight and cell count of five major brain parts: telencephalon, diencephalon,***

***midbrain, cerebellum and brain stem (Fig. 1)**."Line 144-146. We hope that this is clear now.*

L127: In general, I think this is a far better approach since it can tell you what parts of the brain the animal is preferentially investing in. See our goby papers for reference.

Reply 37:

We agree with the referee, and we have now added the White and Brown [6] to our references.

L131: in other words there was an interaction between pop density and performance.

Reply 38:

We have adjusted the sentence accordingly for more clarity. Line 163.

L134: we found similar trade-offs in gobies (https://doi.org/10.1159/000380875)

Reply 39:

We have examined the suggested literature, but it doesn't show that there was a trade-off between telencephalon size and cerebellum size, as the latter was univariably going the same direction as the telencephalon in the studied species (Fig. 4 in White and Brown [7]). Importantly, this study [7] is a comparative study, while here we look at potential trade-offs at the intraspecific level.

L207: we have shown that environmental enrichment in the captive environment also increases cognitive performance (https://doi.org/10.1007/s10071-018-1225-8)

Reply 40:

We thank the reviewer for turning our attention to this paper. We have now added the relevant reference to the text.

L209: this is a bit of a chicken and the egg argument. Given what we know about how incredibly plastic the fish brain is, it seems highly likely to me that fish will only develop enough brain capacity to solve local problems. Which is a slightly different argument than the one you pose here. In other words, there must be a need for adaptive plasticity to generate the brain capacity to cope with that need.

Reply 41:

Obviously, we wouldn't know which one drives the other. Only experimental manipulations would solve such an issue. However, we have added the other possible scenario suggested by the reviewer to our argument. Line 234-235.

L221: Indeed, it's a shame you didn't look at connectivity (synapses). Sounds like a future project.

Reply 42:

We agree.

L231: Yes, in fact you are making a pretty dodgy assumption that the only thing that varies between populations is social structure, but in fact there are likely to be other important environmental differences, such as structural complexity. You make mention of this in your introduction but don't acknowledge it here.

Reply 43:

We have now added the environmental enrichment to this argument. Line 260-262.

L244: I guess the question arises here as to whether the reduction in cerebellum is adaptive or is it the "sacrificial lamb"? The animals need to devote more energy (and space) to the forebrain so something has to give.

Reply 44:

This paragraph tries to discuss the apparent trade-off between forebrain size and cerebellum size, and we tried to explain how the cerebellum can give some space to the forebrain without changing its cell numbers. As stated in this paragraph, we don't know what might be driving such trade-offs as we are not aware of such trade-offs in the literature. Line 264-282.

L253: Some of the papers examining brain miniaturization in ants may be of interest here.

Reply 45:

We thank the reviewer for turning our attention to this literature. We now cite the review by Niven and Farris [8] in this section. Line 280.

L274: Do you mean 2.5m either side of the transect?

Reply 46:

*That's correct. We have now added this information to the sentence in question: "**On every transect line, observers recorded the number of adult cleaners on a 5 m width (i.e., 2.5 m on either side of the transect line)**". Line 300-302.*

L309: This section is a little unclear. Did all fish really undertake 200 trials or did they switch to reversal after reaching criteria?

Reply 47:

Fish were exposed to a total of 200 trials (Line 338), as explained in the Methods section.

Based on the reviewers' comments, we have now deleted text that mentions reversal learning since we did not perform our analyses on these data.

The ten low-performers failed the task (Line 356-357), which means they never reached a learning criteria to pass for the reversal. The ten high-performers, on the other hand, all passed the initial phase, and six among them learned the reversal as well.

L326: It sounds like you did not use the reversal data. Is that the case?

Reply 48:

That's correct, and according to the comments and suggestions provided by the reviewers, we have now deleted the text mentioning reversal learning since we did not perform our analyses on these data.

L331: presumably none of the poor performers made it to the reversal phase. Or did they? There is not much point in running reversals if they didn't learn the first task. I wouldn't bother mentioning this, it just takes up space. It would be good to know the total number of trails the fish were exposed to. It may well be that

there was some plasticity due to training (I think Alex Kortschal has investigated this)

Reply 49:

That's correct: none of the low performers made it to the reversal phase as the criterion to be transited to reversal task was to first success in the initial phase (Line 356-357).

*We have now edited this part accordingly. The new sentence reads as follows: "**The ten high-performers all successfully solved the biological market, while the ten low-performers all failed the task**." Line 356-357.*

We have also deleted any text that refers to the reversal phase since this data was not used in the analyses and mainly based on the reviewers' suggestions.

Fish were exposed to a total of 200 trials as explained in the Methods section (Line 338).

According to the study by Fong, …, Kotrschal, and Kolm [3], cognitive tasks did not affect brain plasticity. We have now included this reference to support our findings (Line 153-154).

L364: how unfortunate

Reply 50:

We agree.

L377: This is my preferred method as mentioned previously.

Reply 51:

We agree.

Reviewer #3:

General comments:

This manuscript reports the results of a study aiming to identify interactions between social environment, brain morphology and cognitive performance in natural populations in cleaner fish. Sampling from four different reef sites, and utilizing an ephemeral reward task, individual fish were tested on task performance with both brain morphology and social density being used to predict learning performance. The performance measure was binary, with fish either learning the task within 200 trials or not. The learning criterion was based on performance over 10 trial sessions. While no relationship was found when looking at measures of the midbrain or brain stem, significant interactions were found

involving both the forebrain and cerebellum with social density. Thus a trade-off was found such that those fish that completed the task tended to have larger forebrains and smaller cerebellums if they were in high density environments but the opposite relationship when in low density environments. Among the fish that did not complete the task, no relationship was found between social density and brain morphology. Given these somewhat counterintuitive effects, the authors then examined the ecological relevance of different decisions rules within the ephemeral task as it pertains to social density differences. From this it was concluded that the results suggest that socially competent fish have larger brains, performing the task using the decision rule most adaptive for their social environment (i.e., reach criterion when from high density environments; do not reach criterion within 200 trials if from low density environment).

I find the topic to be of particular scientific merit as there is a definite need for more studies examining the mechanisms driving individual variation in cognition/task performance, particularly in wild populations, as well as studies trying to explore the ecological factors involved. The authors do a nice job of highlighting how this current study expands upon the literature, and stating their predictions. The paper is generally well written though there are some areas where grammar issues/minor typos make it a little difficult to read. Additionally, while I appreciate the authors wanting to be provide as much information about the study as possible and explain rationale (e.g., reduction of Type 1 error) I do think the paper could be streamlined quite a bit to improve overall clarity and readability. See comments below for some examples.

Reply 52:

We hope that the revised manuscript reads better than the previous version while addressing all the reviewers' concerns and incorporating their suggestions

whenever it was appropriate. Regarding the grammar and the English readability, a native English speaker (co-author Y. Emery) had performed thorough proofreading of the text.

Overall, I found the study to be well designed, and given conservation concerns the sample size is understandable. My main concerns have to do with some of the set up in the introduction and with the authors' interpretation of their results which I feel need to be addressed.

Reply 53:

We have tried to address all the reviewers' concerns in the revised manuscript. We hope that the revised manuscript fits the reviewer's expectations.

First, in the introduction the study is set up with discussion of evolutionary comparative analysis and plasticity, but none of this is really addressed by the study itself. We are told nothing about the underlying genetic differentiation of these populations, and as presented it is unclear if the measured variation is actually due to any plastic response.

Reply 54:

We have now added substantial text to the introduction to fix this issue.

Discussion of ontogeny is then continued in the discussion, but again the role of development on brain morphology and task performance is not examined in this study. Even the studies that are referenced do not seem to directly address this concern. I would suggest reframing the study away from these topics unless you feel you can better connect them with
the current study.

Reply 55:

We mention ontogeny and development in the conclusive paragraph (Line 284-287). Here, we suggest these aspects as a potential explanation of the findings given the ecology of the species and more specifically the ecology of this population at Lizard Island. Also, we have now added substantial text to the introduction that support our arguments and conclusions. We hope that these changes help to better connect such aspects with the current study.

The second issue has to do with the interpretation of the interaction between brain morphology (forebrain and cerebellum) and social environment. While there is a significant interaction effect indicating that the slopes of learners and non-

learners differ, alone this doesn't say anything about how these groups differ at different densities? Were any post-hoc contrasts done? Without showing these contrasts, the authors need to watch out for making comparisons between groups (e.g., Lines 151-152), and be a little careful about how they relate these findings to their initial predictions and in their justification for setting up the social competence narrative.

Reply 56:

*Please refer to **Reply 7** where we address the very same concern.*

The final issue has to do with the social competence framework itself. While the rationale is clearly articulated for why you would expect to see social competence and task performance be in line with one another at higher densities, why you would expect worst performance from socially competent fish from low social densities conditions is still unclear to me. How is getting less food (consequence of incorrect trials) optimal in this case. Also, if visitors have no choice in low densities and so have to stick around, is this task really relevant to these conditions? Visitor cues should simply be irrelevant, but this wouldn't mean it would be optimal to not follow cues during the ephemeral reward task. Particularly if you want to argue that larger brained fish that are presumably more "intelligent" or socially competent are better able to adapt plastically to local conditions (lines 210-211) then I think you would expect them to adapt to the learning conditions of the task faster.

Reply 57:

We have now added a substantial amount of text to the introduction to explain in details why not prioritising a visitor client at low densities can be viewed as a an optimal strategy (i.e., social competence).

Also, the first paragraph of the discussion explicitly states that the biological market was probably not adequate and well adapted for all the fish. Further research with varying scenarios of the biological market are thus needed.

While it is interesting that you see a significant difference in brain morphology when you looked to examine social competence, more work needs to be done to build the argument that manipulating the data in this way is warranted biologically. While I would like to see all of these concerns addressed before I would consider this study ready for publication, given the overall conclusions from the paper I believe addressing this final concern is the most critical.

Reply 58:

In the new revision, we have added a paragraph to the introduction to better explain the logic of social competence and how this hypothesis can explain the documented variation in cleaner fish behaviour within the same population.

We hope that the extra information provided about cleaner fish ecology in the introduction provides a strong argument for encoding performance from "high" and "low" to "optimal" and "not-optimal" strategies, depending on the ecology of the studied individuals.

Detailed comments:

Lines 80-81: Unclear if this is saying these differences have evolved amongst populations or are due to developmental plasticity

Reply 59:

This sentence has been deleted in the revised version of the manuscript.

Line 94: A little hard to tell from the figures but it seems like success task success was generally similar across social density groups? Was this true? Maybe summarize number of successful fish across density groups in the Results?

Reply 60:

We agree that the information was missing in the main text. We have now added detailed information about the sampled fish in a Supplementary table (S1).

Line 101: Why only females? Sex effects?

Reply 61:

*We have now added a few sentences to the Methods section to explain the use of female cleaners in the current study: "**Only female cleaners were collected for this study since the population is female biased (Robertson 1973), and also to facilitate the comparison to previous studies on female cleaner fish (Salwiczek et al. 2012; Wismer et al. 2014; Triki et al. 2018, 2019b).**" Line 309-311.*

Line 106: This wording is confusing. On my initial read it seemed to imply 20 individuals from each of 4 sites (so 80 total)

Reply 62:

*We made it clearer in the text, the sentence reads as follows: " **We then caught and tested a total of 40 female cleaners**…". Line 136-137.*

Paragraph starting with line 151: Initially this interpretation of results seem better suited for the discussion. I understand that this is needed to set up the follow up analysis, but is there maybe a better way to set this up in the intro so that it doesn't seem to come out of nowhere in the middle of the results? Feels out of place here.

Reply 63:

We agree that this text fits better in the introduction instead of the results section. We have now moved this text to the introduction.

Lines 151-152: As presented the stats don't say anything about the comparisons across performers and nonperformers being significant. I understand the authors simply point to "relative" differences of the observed fish but need to be careful about overinterpreting results about group comparisons.

Reply 64:

Please refer to ***Reply 7*** *where we address the very same concern.*

Line 170: Why would it be "optimal" to ignore visitors and get less food in low density context? Are you suggesting costs to identifying visitors?

Reply 65:

We provide a more detailed explanation to our arguments about optimal and not-optimal strategies in the revised introduction, and we address these very concerns in an explicit way.

Lines 178-182: I found this sentence awkwardly written. I would suggest rewriting it.

Reply 66:

*We have rewritten this sentence for more clarity. It reads as follows: "**To do so, we set a critical threshold of cleaner population density that determines which decision-rule is locally adaptive, which was set at 1.5 cleaner fish per 100 m2. We estimated this threshold from the study by Triki et al. (see Fig. 2c in Triki et al. 2019)**." Line 189-192.*

Lines 209-211: But then wouldn't the bigger brained fish show an ability to adapt to the test environment? Instead the smaller brained fish are getting more food.

Reply 67:

Our study suggests that the decision-rules applied by cleaner fish in the lab-based task stem from experience in their natural habitats (First paragraph of the Discussion).

According to behavioural observations by Triki et al. [9], the occasions in which a cleaner faces a visitor and a resident seeking cleaning service simultaneously can range between 62 to 850 occasions per day (an estimate for 11 hours of cleaning activity). This is still far from our 20 trials per day. It suggests that recorded performance during 200 trials (10 days of testing) is indeed reflecting the learned rules of the cleaners.

In order to learn new rules in lab settings, probably thousands of trials would be needed and it might depend strongly on the brain morphology of these fish.

We have now added this argument to the first paragraph of the discussion. Line 213-221.

Line 217: With the slightly different section order of this publication I would reference your methods here so the reader knows that the reason for this missing data will be explained there.

Reply 68:

Following the reviewer's suggestion we refer to the Methods section here. Line 241.

Line 232: "captures" instead of "capture"

Reply 69:

We corrected this word. Line 258.

Line 304: 200 trials per fish for 10 days could be interpreted as 2000 trials total for each fish. Maybe reword.

Reply 70:

*We added information to make it clear that every fish had a maximum 200 trials. The test period extended over ten days. The new sentence reads as follows: "**For ten consecutive days, we ran a total of 200 trials per fish. We tested every fish in two sessions (i.e., one session constituted 10 trials) a day, with one session in the morning and one in the afternoon.**" Line 338-340.*

Lines 311-312: In what way were things counterbalanced? Between trials, between fish? I assume between fish such that the pattern and status of the plates differed between fish but were consistent for that fish over all of their trials, but was side also consistent for each fish? In other words was the "visitor plate" always on the right for Fish A?

Reply 71:

*We see how this can be confusing. We have now tried to clarify this part of the methods of testing. The edited sentence reads as follows: "**Also, the decoration (i.e., vertical pink stripes or horizontal green stripes) and the status of the plates (i.e., visitor or resident) were counterbalanced between fish, while the spatial location (i.e., left or right) was randomised and counterbalanced between trials. That is, no more than three trials in a row for the same spatial location with a 50:50 ratio for a plate to be presented on the left or right***

side." *Line 344-349*.

Line 344: Maybe reorder your figures for that Fig 1 isn't being referenced after other figures.

Reply 72:

We have now made sure that figures' referenced in the main text are in a correct order.

Lines 363-366: This should be referenced above in the discussion, or maybe moved to the discussion when you talk about not having neuron numbers.

Reply 73:

We have now reference to this in the discussion so the reader can understand that there is a technical explanation for the missing data in the Methods section. Line 241.

Lines 386-387: Example of a line that could be cut since it is redundant with text found in the results section.

Reply 74:

We have now deleted this sentence from the results section, while keeping it in the Methods section (Line 397-402) as it explains the rationale of the scaling method used to generate the statistical outcomes and figures.

Line 414-415: What were these behavioral observations for? The audience effect mentioned above? Feel this can should be removed along with discussion of audience effects if not used for the analysis of this study. Similarly I think mentions of the reversal learning phase could also be removed.

Reply 75:

We thank the reviewer for pointing out the mismatch in the text. Initially, we had behavioural observations incorporated from the study by Triki et al. (2019). However, we aimed afterwards to simplify this part by referring directly to that study.

We have now deleted this text.

Regarding the audience effect, we have now shortened that paragraph to become:

"Before the ephemeral reward task, all cleaners had been tested in an audience effect task. Unfortunately, this task did not generate the desired effect of having enough variation to classify individuals as high- or low-performers as they all performed poorly in this task (see Supplementary Methods)." *Line 317-321.*

This helped with being transparent about the fact that all fish had been subject to another task. Nevertheless, the details are not in the main text but kept as a supplementary material.

Cited Literature:

1. Eckert, G. J. Estimates of adult and juvenile mortality for labrid fishes at One Tree Reef, Great Barrier Reef. *Marine biology* **95**, 167–171 (1987).
2. Triki, Z., Levorato, E., McNeely, W., Marshall, J. & Bshary, R. Population densities predict forebrain size variation in the cleaner fish *Labroides dimidiatus*. *Proceedings of the Royal Society B: Biological Sciences* **286**, 20192108 (2019).
3. Fong, S., Buechel, S. D., Boussard, A., Kotrschal, A. & Kolm, N. Plastic changes in brain morphology in relation to learning and environmental enrichment in the guppy (*Poecilia reticulata*). *The Journal of Experimental Biology* **222**, jeb200402 (2019).
4. Thornton, A. & Lukas, D. Individual variation in cognitive performance: developmental and evolutionary perspectives. *Philosophical Transactions of the Royal Society B: Biological Sciences* **367**, 2773–2783 (2012).
5. Varela, S. A. M., Teles, M. C. & Oliveira, R. F. The correlated evolution of social competence and social cognition. *Functional Ecology* **0**, 1–12 (2019).
6. White, G. E. & Brown, C. Variation in Brain Morphology of Intertidal Gobies: A Comparison of Methodologies Used to Quantitatively Assess Brain Volumes in Fish. *Brain, Behavior and Evolution* **85**, 245–256 (2015).
7. White, G. E. & Brown, C. Microhabitat Use Affects Brain Size and Structure in Intertidal

Gobies. *Brain, Behavior and Evolution* **85**, 107–116 (2015).

8. Niven, J. E. & Farris, S. M. Miniaturization of Nervous Systems and Neurons. *Current Biology* **22**, R323–R329 (2012).

9. Triki, Z. *et al.* Biological market effects predict cleaner fish strategic sophistication. *Behavioral Ecology* **30**, 1548–1557 (2019).

Reviewer #1 (Remarks to the Author):

The present study “Trade-off between forebrain and cerebellum size is associated with cleaner fish cognitive performance as a function of population density” by Triki and colleagues takes advantage of the unique biology of cleaner fish to explore associations between brain characteristics, ecological context, and performance of a complex task. I find the innovative utilization of such a fascinating model system compelling. The results interesting and complex. I reviewed a previous version of the manuscript

I thank the authors for their responses to my previous concerns. I find the manuscript much improved and have only very minor comments remained.

I realize now some of my confusion reading the previous version of the manuscript came from a misunderstanding concerning the relationship between sampling of high- and low- performers and then ‘re-grouping’ these same individuals into ‘optimal’ versus ‘non-optimal’. This design is very nice and I think including a more explicit explanation of this approach and why it was taken would help readers like myself who are unfamiliar with the system, as well as making the choice of the social competence framing more clear.

Figures 2 & 3: The x-axis is missing a label.

Reviewer #2 (Remarks to the Author):

The authors have done an excellent job responding to comments. The revised version is superior to the previous one. I have no further comments.

Small note: the font in ref 44 is upper case for some reason.

Reviewer #3 (Remarks to the Author):

I think the authors have a done a nice job of addressing my major concerns, particularly in their explanation of a possible mismatch between task performance and social competence in the low density environment. I am satisfied with the edits made and now recommend the paper for publication. I do have a few extra edits that the authors may want to consider:

Lines 76-78: If you are going to highlight the point that fish from high density areas are usually better performers than low density fish in previous studies it seems a little weird that it isn't directly addressed later in your analysis. I understand you can't look at the fish that you sacrificed since they were chosen based on their performance, but what about the 40 fish sampled overall?

Lines 120-122: I buy this explanation, but do we know how the fish are distinguishing between locals and visitors? Is it based on species differences?

Line 122-128: The way the sentences before these are written it doesn't really make the reader expect a list of arguments, so then going into a sentence that starts with "First,..." seemed a little awkward to read.

Line 136: I would move sentence about why only females from the Methods to here in the introduction.

Lines 149-154: I think the authors have done a good job of incorporating social competence into the

new intro. However, I think the alternative predictions of the social competence hypothesis should be stated here in contrast to the prediction that that high performers should just have larger brains.

Line 224: I think around here you could add something like "Therefore leading to highly socially competent (i.e., more intelligent) fish possibly performing poorly on the task." Just to help drive the logic all the way home.

Line 248-250: Again, this makes sense for the 20 sacrificed fish, but what about the 40 fish. Those were randomly sampled right?

We thank the reviewers for their valuable comments and suggestions. Please see below the detailed response for each point in turn.

Reviewer #1 (Remarks to the Author):

The present study "Trade-off between forebrain and cerebellum size is associated with cleaner fish cognitive performance as a function of population density" by Triki and colleagues takes advantage of the unique biology of cleaner fish to explore associations between brain characteristics, ecological context, and performance of a complex task. I find the innovative utilization of such a fascinating model system compelling. The results interesting and complex. I reviewed a previous version of the manuscript

I thank the authors for their responses to my previous concerns. I find the manuscript much improved and have only very minor comments remained.

I realize now some of my confusion reading the previous version of the manuscript came from a misunderstanding concerning the relationship between sampling of high- and low- performers and then 're-grouping' these same individuals into 'optimal' versus 'non-optimal'. This design is very nice and I think including a more explicit explanation of this approach and why it was taken would help readers like myself who are unfamiliar with the system, as well as making the choice of the social competence framing more clear.

Figures 2 & 3: The x-axis is missing a label.

Reply 1:

We have now added the missing labels to Figure 2 & 3.

Reviewer #2 (Remarks to the Author):

The authors have done an excellent job responding to comments. The revised version is superior to the previous one. I have no further comments.

Small note: the font in ref 44 is upper case for some reason.

Reply 2:

We have adjusted the font of ref 44.

Reviewer #3 (Remarks to the Author):

I think the authors have a done a nice job of addressing my major concerns, particularly in their explanation of a possible mismatch between task performance and social competence in the low density environment. I am satisfied with the edits made and now recommend the paper for publication. I do have a few extra edits that the authors may want to consider:

Lines 76-78: If you are going to highlight the point that fish from high density areas are usually better performers than low density fish in previous studies it seems a little weird that it isn't directly addressed later in your analysis. I understand you can't look at the fish that you sacrificed since they were chosen based on their performance, but what about the 40 fish sampled overall?

Reply 3:

As it stands the entire ecosystem at Lizard Island is out of balance since the major perturbations (cyclones in 2014/15 and coral bleaching in 2016), which means that cleaner fish do not perform that predictably any more at the various study reef sites. For that very reason, we adjusted our experimental design of this study to meet the

new challenges. A previous paper by Triki et al.[1] *had already failed to document consistent differences in performance across reef sites. Therefore, we decided to focus on high- and low-performance across sites for the current study. We point out the effect of perturbations in the new revision.*

The data for the performance in the biological market of the 40 female cleaners from all four sites are accessible via the Figshare link).

Lines 120-122: I buy this explanation, but do we know how the fish are distinguishing between locals and visitors? Is it based on species differences?

Reply 4:

*We have now added explicitly to the text that cleaners do distinguish between residents and visitors. We refer to other papers for details on that. In the text we wrote: "**Several studies show that cleaners are able to distinguish resident client species from visitor species as they treat them differently, though the precise mechanisms are not known**."*

Line 122-128: The way the sentences before these are written it doesn't really make the reader expect a list of arguments, so then going into a sentence that starts with "First,..." seemed a little awkward to read.

Reply 5:

*We have now adjusted the sentence accordingly. We have added: "**... for several reasons**" before "**First,...**".*

Line 136: I would move sentence about why only females from the Methods to here in the introduction.

Reply 6:

We moved that sentence from the Methods to the Intro as suggested by the reviewer.

Lines 149-154: I think the authors have done a good job of incorporating social competence into the new intro. However, I think the alternative predictions of the social competence hypothesis should be stated here in contrast to the prediction that that high performers should just have larger brains.

Reply 7:

We thank the reviewer for the suggestion. We already have a similar statement later on in the text (starting from Line 194). Here we explain that now we switch to testing social competence hypothesis predictions instead of using high- and low-performance. We prefer to keep it as is to avoid redundancy in the text.

Line 224: I think around here you could add something like "Therefore leading to highly socially competent (i.e., more intelligent) fish possibly performing poorly on the task." Just to help drive the logic all the way home.

Reply 8:

We have added the suggested sentence to the paragraph in question. It reads now more clearly in this part.

Line 248-250: Again, this makes sense for the 20 sacrificed fish, but what about the 40 fish. Those were randomly sampled right?

Reply 9:

That's correct. The 40 fish were sampled randomly. Please see Reply#3 for our response to a similar comment.

Cited literature:

1. Triki, Z., Wismer, S., Levorato, E. & Bshary, R. A decrease in the abundance and strategic sophistication of cleaner fish after environmental perturbations. *Glob Change Biol* **24**, 481–489 (2018).
